# Semantic Processing in Healthy Aging and Alzheimer’s Disease: A Systematic Review of the N400 Differences

**DOI:** 10.3390/brainsci10110770

**Published:** 2020-10-23

**Authors:** Marilyne Joyal, Charles Groleau, Clara Bouchard, Maximiliano A. Wilson, Shirley Fecteau

**Affiliations:** 1 CERVO Brain Research Center, Institut Universitaire en Santé Mentale de Québec, Centre Intégré Universitaire de Santé et de Services Sociaux de la Capitale-Nationale, 2601 Chemin de la Canardière, Québec, QC G1J 2G3, Canada; marilyne.joyal.1@ulaval.ca (M.J.); charles.groleau.1@ulaval.ca (C.G.); clara.bouchard.1@ulaval.ca (C.B.); Maximiliano.Wilson@fmed.ulaval.ca (M.A.W.); 2 Faculty of Medicine, Université Laval, Pavillon Ferdinand-Vandry, 1050 Avenue de la Médecine, Québec, QC G1V 0A6, Canada

**Keywords:** semantic processing, healthy aging, Alzheimer’s disease, N400, systematic review

## Abstract

Semantic deficits are common in individuals with Alzheimer’s disease (AD). These deficits notably impact the ability to understand words. In healthy aging, semantic knowledge increases but semantic processing (i.e., the ability to use this knowledge) may be impaired. This systematic review aimed to investigate semantic processing in healthy aging and AD through behavioral responses and the N400 brain event-related potential. The results of the quantitative and qualitative analyses suggested an overall decrease in accuracy and increase in response times in healthy elderly as compared to young adults, as well as in individuals with AD as compared to age-matched controls. The influence of semantic association, as measured by N400 effect amplitudes, appears smaller in healthy aging and even more so in AD patients. Thus, semantic processing differences may occur in both healthy and pathological aging. The establishment of norms of healthy aging for these outcomes that vary between normal and pathological aging could eventually help early detection of AD.

## 1. Introduction

Semantic knowledge refers to our general knowledge about the world. Semantic processing or semantic cognition enables us to use this knowledge in order to understand the meaning of words, recognize objects in the environment and perform cognitive tasks, such as identifying the presence or absence of links between concepts [1,2,3].

Patients with Alzheimer’s disease (AD) are known to display semantic deficits [4,5]. Nevertheless, it is yet unclear whether these semantic deficits are specific to AD or common to both pathological and healthy aging. Indeed, in healthy aging, semantic knowledge has long been considered intact since it increases with age [6,7]. However, recent studies suggest that semantic processing may decline with age [8]. Some studies observed decreased accuracy in semantic tasks which require an explicit use of semantic knowledge in elderly as compared to younger adults [9,10,11], whereas other studies found similar accuracy for both age groups (e.g., [12,13,14]). This raises the consequential question of whether such potential decline in semantic processing in healthy aging is similar or not (and, if so, to which extent) to the observed deficits in AD.

One of the brain correlates known to be associated with semantic processing is the N400 event-related potential (ERP). Specifically, the N400 amplitude and peak latency represent the levels and timing of semantic activity in long-term memory that is elicited by a given stimulus relative to baseline states of activity [15]. Notably, stimuli for which semantic information has been recently preactivated through feature overlap with associated stimuli typically induce a reduction in the N400 amplitude in comparison to a control condition (e.g., with nonassociated stimuli). There is also the N400 effect, which is an index of semantic processing calculated as the amplitude difference between two semantic conditions (e.g., semantically associated and nonassociated stimuli; [15]). It has been reported that patients with AD showed decreased N400 effects as compared to healthy elderly adults [16,17,18] and that healthy elderly adults displayed decreased N400 effects as compared to young adults (e.g., [19,20,21]). Some studies indicated that the reduction in the N400 effect is due to smaller (i.e., less negative) N400 amplitudes for semantically unrelated or incongruent stimuli. This was found in AD patients [22,23] and elderly adults [10,24], although larger N400 amplitudes were also found for semantically congruent or predictable stimuli in these populations [25,26]. It remains unclear whether the N400 effect differences observed in healthy and pathological aging are due to changes in the processing of stimuli that are highly associated (e.g., rabbit—carrot), weakly associated (e.g., bird—carrot) or both. In sum, it appears important, when studying semantic processing, to consider several semantic related measures (e.g., behavioral accuracy and response time; N400 amplitude, latency, and effect), as well as the studied semantic conditions (e.g., high vs. weak semantic association).

The goal of this systematic review was two-fold: (1) to investigate whether behavioral performance of semantic processing in healthy elderly is intact, different or impaired as compared to young adults, and (2) to identify whether the well-characterized semantic deficits seen in AD reflect normal aging or are specific to AD. In order to address these goals, we compared behavioral (accuracy and response time) and N400 related measures (N400 amplitude, latency, and effect) during tasks that involve semantic processing between healthy older and younger adults, as well as between AD and healthy elderly adults. We postulate that if healthy elderly adults display similar behavioral performance as compared to that of younger adults, then semantic processing can be considered to be intact in healthy aging. This would be the case even if behavioral performance differs from the N400 related measures. Behavioral performance may rely on other brain substrates than the N400 related measures, indicating potential brain reorganization [27]. If healthy elderly adults display different behavioral performance as compared to that of younger adults but similar to that of AD individuals, this would indicate that what we see in AD is not specific to AD and reflects normal (albeit exacerbated) aging. If healthy elderly adults display different behavioral performance as compared to that of younger adults and different to that of AD, this will indicate that what we see in AD may be specific to AD and differ from normal aging. Ultimately, the identification of the semantic conditions (e.g., highly or weakly associated stimuli) that may differ between normal and impaired aging could allow us to detect early and better monitor the evolution of pathological aging.

## 2. Materials and Methods

We conducted this review following PRISMA guidelines [28]. The PRISMA checklist is provided in Supplementary Material (Table S1).

### 2.1. Literature Search

We conducted a systematic database search in PubMed, PsycINFO and Embase on articles published before 7 February 2020 using both controlled vocabulary and keywords terms related to concepts of “N400” and “Aging” or “Alzheimer’s disease (AD)”. Detailed search terms are provided in Supplementary Materials (Table S2) for each database. This database search resulted in a total of 10,775 abstracts for potential inclusion.

### 2.2. Selection Criteria

**Figure 1:**
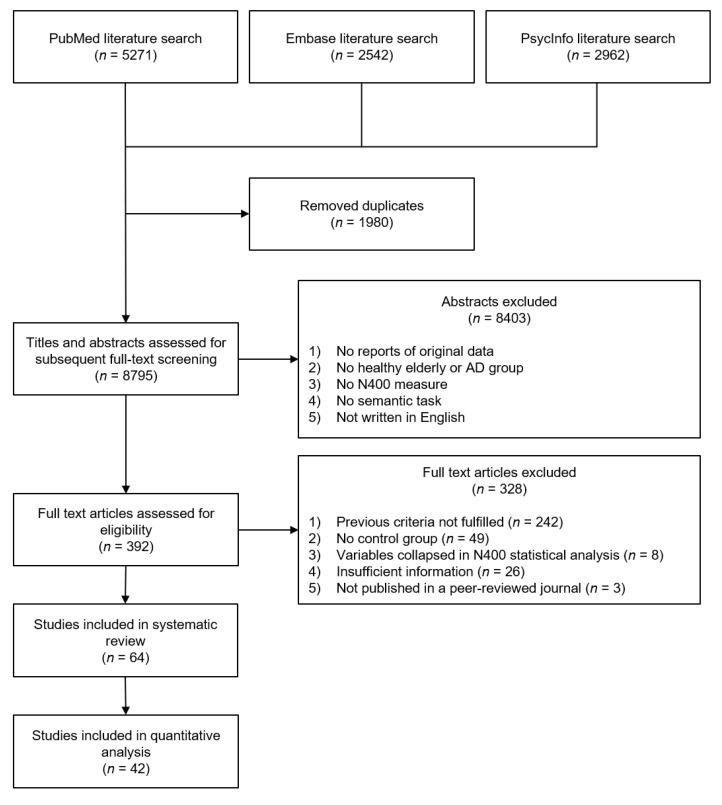
Schematic overview of the study selection procedure in this systematic review.

In a first phase of selection, two independent reviewers (MJ, CG) read titles and/or abstracts and excluded articles (a) not reporting original data (e.g., reviews, meta-analyses, opinion letters), (b) not assessing a group of healthy older participants or a group of participants with a diagnosis of AD, (c) not measuring the N400 event-related potential, (d) not implementing a semantic task with the manipulation of a semantic variable, and (e) not written in English. This left a total of 392 original studies. Two reviewers then performed a full text screening (MJ, CB) and further excluded 328 of the 392 studies due to the preceding reasons or if the studies (f) had no statistical comparison with a control group (i.e., a group of younger participants to be compared with the healthy elderly group, or a group of healthy elderly participants to be compared with an AD group), (g) presented different conditions collapsed in the N400 statistical analysis, making it impossible to isolate the effects of the semantic variable manipulated, (h) had insufficient information, preventing us from assessing all eligibility criteria, and (i) were not published in peer-reviewed journals, since unpublished studies tend to show lower methodological quality than peer-reviewed articles [29,30]. In case of disagreement, a third reviewer (MW) performed the screening to help reach a final decision. This procedure led to the inclusion of 64 studies in the review (Figure 1). In order to perform the statistical analyses of the quantitative review, we needed to extract mean values (i.e., group means of performance) for experimental outcomes of interest from the articles in the review. These mean values were reported in 42 studies out of the 64 retained for the present review. These studies were thus those included in the quantitative analysis. The 64 articles were included in the qualitative review.

### 2.3. Quantitative Analysis

We extracted mean amplitudes and latencies of the N400, as well as accuracy scores and mean response times (RTs) in semantic judgment tasks, for each group and semantic association condition. We extracted data from the text, tables or figures of the articles. We tested potential interactions between age and semantic association by entering each outcome reported by ≥10 studies into separate univariate ANOVAs with fixed factors Group (older vs. younger or AD vs. aged-matched controls) and Semantic association (highly vs. weakly associated) and the random factor study. Of note, we pooled together congruent, related, associated, matched and predictable target stimuli in the high association condition, as they share semantic links with the preceding prime stimuli. Consequently, we included incongruent, unrelated, nonassociated, mismatched and unpredictable target stimuli in the weak association condition. We also included the sample size as a weight variable in the ANOVAs, as previously done by Ali et al. [31]. In case of a significant interaction, we performed simple effects tests. We also extracted mean amplitudes and latencies of the N400 effect for each group (since N400 effect measures are typically calculated from the difference wave between two semantic conditions, there is no factor of semantic association). If the N400 effect amplitudes were not available but N400 amplitudes were reported, we calculated the N400 effect for each group by subtracting the N400 amplitude for highly associated stimuli from the amplitude for weakly associated stimuli. When the amplitude and latency of the N400 effect could be extracted or calculated for ≥10 studies, we performed separate univariate ANOVAs with the fixed factor Group (older vs. younger or AD vs. aged-matched controls), the random factor study and the sample size as weight variable.

When values were available for multiple age groups in a single study, we extracted values from the older and younger adult groups. Additionally, when there were multiple conditions (e.g., two different types of semantic links), we chose only one representative condition (e.g., “nonmetaphorical semantic link” rather than “metaphorical semantic link”) for the quantitative analysis in order to give each study the same number of experimental conditions. Furthermore, when values were available for more than two levels of semantic links (e.g., best completion vs. related/sense vs. related/nonsense vs. unrelated/nonsense), we extracted mean values of the conditions with the strongest (e.g., best completion) and weakest (e.g., unrelated/nonsense) levels of semantic links. Statistical analyses were performed using SPSS 25 (SPSS Inc., Chicago, IL, USA). We considered *p*-values <0.05 as statistically significant.

### 2.4. Qualitative Review

We collected data relative to study design, participants, materials and procedures, as well as the behavioral and N400 outcomes on semantic tasks. Specific attention was given to key elements of materials and procedures that should be reported in EEG studies [32]. When the information was not clearly identifiable, we considered it as not reported. We only reported results obtained from statistical comparisons between groups of participants and simple effects tests.

## 3. Results

Please refer to Table A1 and Table A2 provided in the appendix for the main characteristics of the studies included in this review for the healthy and AD groups, respectively.

### 3.1. Study Design

All articles reviewed described observational studies, mostly case-control studies (*n* = 56). Some were observational studies with a single group of older participants (*n* = 6), but they referred to another article in which the same materials and procedures were used with a group of young participants, from which we could extract the data. One study can be characterized as a proof of concept study and another one as a correlational study.

### 3.2. Participants

The 64 retained articles included a total of 1097 healthy elderly participants (mean age = 67.7 years old, *SD* = 5.9), 1060 healthy younger participants (mean age = 23.4 years old, *SD* = 5.6) and 181 AD participants (mean age = 71.7 years old, *SD* = 8.1). These samples were respectively composed of 53.7%, 53.2% and 49.6% of females. The mean number of years of education was reported in 33 studies (older = 14.6 years, younger = 15.2 years, AD = 13.2 years). In some studies, groups were matched for age (AD vs. controls; *n* = 6), sex (*n* = 5) and education (*n* = 16). AD participants were at a mild or moderate stage of the disease (this information was unavailable in four studies). AD participants had mean scores at the Mini-Mental State Examination (MMSE; [33]) ranging from 16.5 to 25.1 (mean = 21.6, *SD* = 2.9). None of the studies reported any sample size calculation, but two reported some justification of the sample size (e.g., greater number of older participants to better equate power in the two samples). Twenty-one studies reported the exclusion of participants. At least 47 elderly participants, 17 AD participants and 22 younger participants were excluded, mainly due to excessive artefacts in electroencephalographic (EEG) data.

### 3.3. Materials and Procedures

#### 3.3.1. Stimuli

In most of the studies, stimuli were sentences with the target word in the final (*n* = 24), medial (*n* = 6) or varying positions (*n* = 1). Other experimental tasks involved word pairs (*n* = 15), picture pairs (*n* = 5), or word-picture/picture-word pairs (*n* = 4). Other studies included phrases followed by a target word (*n* = 5), series of five words (*n* = 1), word triplets (*n* = 2), single words (*n* = 3) or number pairs (*n* = 1). Target stimuli were either presented visually (*n* = 52), auditorily (*n* = 11) or both (*n* = 1). Word stimuli were in English (*n* = 36), Dutch (*n* = 6), Chinese (*n* = 5), French (*n* = 2), Swedish (*n* = 2), German (*n* = 2), Hungarian (*n* = 1), Finnish (*n* = 1), Korean (*n* = 1) or in two different languages within the same task (*n* = 2). From the 57 studies which involved an experimental task with words as targets, 34 matched stimuli across some conditions for at least one psycholinguistic variable (e.g., word length, frequency). Of note, psycholinguistic characteristics of words were reported in nine additional studies, although no statistical tests were performed for the matching of stimuli.

#### 3.3.2. Experimental Tasks

The included studies used various tasks, including semantic judgment (i.e., making a decision about the meaning of stimuli; *n* = 35), reading (*n* = 19), listening (*n* = 5), lexical decision with semantic priming (*n* = 3), recognition memory (*n* = 1) or a conceptual combination and frequency-comparison tasks (*n* = 1). The number of semantic conditions in these tasks varied from 2 to 6. Almost all studies (n = 63) measured semantic association, by means of at least one of the following factors: stimuli congruence (congruent vs. incongruent, *n* = 30), relatedness (related vs. unrelated, *n* = 14), predictability/expectancy (expected vs. unexpected, *n* = 12), matching (match vs. mismatch, *n* = 5), association (high vs. low, *n* = 4), plausibility (plausible vs. implausible, *n* = 2), correctness (correct vs. semantic anomaly, *n* = 1) or synonymy (synonym vs. nonsynonym, *n* = 1). Some studies measured the influence of other semantic variables: semantic link (metaphorical or idiomatic vs. nonmetaphorical or literal *n* = 2; category vs. antonymy, *n* = 2), concreteness (concrete vs. abstract, n = 2), typicality (high vs. low, *n* = 1), category level (superordinate vs. basic vs. subordinate, *n* = 1) or semantic selection demand (high vs. low, *n* = 1). The number of trials per condition ranged from 22 to 256 (mean = 70.1, *SD* = 48.3). In 47 studies, targets were presented for a fixed amount of time, ranging from 100 to 2000 ms. In other studies, the presentation time of targets varied between trials or conditions (*n* = 5) or targets remained on the computer screen until the participant responded or a time limit was reached (*n* = 4). Experimental tasks required a response from the participants at each trial in 47 studies and on filler trials in one study. In other studies, no response was required during the task, but in most cases participants had to perform a recognition or recall memory test between blocks (*n* = 4) or after the end of the task (*n* = 9). Of note, whether participants had to respond or not was unclear in one study. From the 47 studies which required a response during the task, participants had to respond by button press (*n* = 37) or verbally (*n* = 8). Response modality was not reported in two studies. Of note, 25 studies designed their task so participants’ responses were delayed primarily to reduce movement artefacts.

#### 3.3.3. EEG Recording

The number of electrodes used for the EEG recordings varied from 3 to 128 (mean = 27.4, *SD* = 21.5). Fifty studies reported the name of the EEG system they used and 27 reported the name of the EEG software. Impedances during recordings were kept below 5 kΩ in most studies (*n* = 32), but 21 studies did not report this information. Data were referenced to the average of both mastoids (n = 34), linked earlobes (*n* = 12), the nose (*n* = 5), the right mastoid (*n* = 4), the left mastoid (*n* = 3), the right earlobe (*n* = 1) or the average of all electrodes (*n* = 4). The reference electrodes were not mentioned in one study. Sampling rates ranged from 100 to 1024 Hz (mean = 347.6, *SD* = 245.5) but were not reported in three studies.

#### 3.3.4. Preprocessing

EEG data were high-pass filtered in the majority of studies (*n* = 47), with cut-off values between 0.01 and 1.25 Hz (mean = 0.23, *SD* = 0.30). EEG data were low-pass filtered in all studies, mostly with cut-off values of 100 Hz (*n* = 23) and 30 Hz (*n* = 15), although the cut-off varied between 8 and 300 Hz (mean = 73.2, *SD* = 49.9). Epoch duration ranged from 750 to 2200 ms (mean = 1137.5, *SD* = 315.9) after target onset. Epochs were computed to a relative prestimulus baseline (mean = 151.1 ms, *SD* = 115.9) in 44 studies. All studies removed or corrected EEG artefacts, using one or more of the following methods: correction of ocular artefacts (*n* = 31), removal of trials based on thresholds (*n* = 29), visual inspection (*n* = 23) or independent component analysis (*n* = 9). Of note, methods used to remove contaminated trials were not described in eight studies. Twenty-nine studies reported the number or percent of trials removed and 12 studies reported the mean or minimum number of remaining trials.

#### 3.3.5. Statistical Analysis

Reviewed studies measured mean amplitude (*n* = 54), peak amplitude (*n* = 14), peak latency (*n* = 29), fractional area latency (*n* = 5), individual ERP topographies (*n* = 2) or electrical field strength (*n* = 1) of the N400 or N400 effect (i.e., difference wave between two semantic conditions, such as congruent vs. incongruent). Time-windows for the analysis of the N400 varied, with a starting point between 200 and 800 ms and an ending point between 276 and 1000 ms. Twenty-five studies used a single time-window for all groups of participants and experimental conditions, whereas time-windows varied in other studies. The time-window was not reported in two studies. A total of 36 studies provided a justification for the choice of the time-window (based on visual inspection or data driven, *n* = 19; based on previous studies, *n* = 10; or both, *n* = 6). The number of electrodes considered for the analyses of the N400 varied from 1 to 128 (mean = 14.6, *SD* = 16.5). Data were either analyzed for each individual electrode (*n* = 36), the average of a single group of electrodes (*n* = 7), or multiple regions of interest (*n* = 21). Forty studies provided some justification for the selection of the electrodes included in the statistical analyses. The justifications were mostly based on previous studies (*n* = 24) or on visual inspection/data driven (e.g., analyzed electrodes with the largest peak amplitudes; *n* = 13). Most studies reported ANOVAs to analyze the effects of age/group or semantic condition on the N400 (*n* = 50). Other statistical tests used were linear mixed-effect models (*n* = 5), t-tests (*n* = 6), multivariate analysis of variance (*n* = 4), regression analysis (*n* = 2), analysis of covariance (*n* = 1), topographic analysis of variance and randomization tests (*n* = 1) or Kruskall–Wallis tests (*n* = 1). From the 56 studies which performed multiple comparisons, 21 reported the use of a correction method to control for type 1 error (Bonferroni, *n* = 8; Tukey, *n* = 5; False discovery rate, *n* = 2; Newman-Keuls, *n* = 2; Fischer’s least significant difference, *n* = 2; Scheffé, *n* = 1; Jackknife correction, *n* = 1). From the 53 studies which performed repeated measures analyses, 42 used an epsilon correction (Greenhouse-Geisser correction, *n* = 29; Huynh–Feldt, *n* = 13).

In what follows, we present the behavioral and N400 results of the present systematic review for healthy participants followed by those of Alzheimer’s disease patients. For the sake of simplicity, for each section, we produced a general summary of the results, followed by the quantitative analyses and the qualitative review. We will only present the results of the main effects of interest age/group and semantic association and their interaction. For a more detailed summary of interactions or age/group effects related to other semantic variables (e.g., concreteness), we refer the reader to Table A1 and Table A2.

### 3.4. Behavioral and N400 Outcomes on Semantic Tasks

#### 3.4.1. Behavioral Accuracy

For the healthy elderly, the quantitative results of the studies grouped together show lower accuracy in older than younger adults for semantic association, regardless of the stimuli, but the qualitative review indicates that only a few studies found significant main effects of age. For AD patients, the qualitative review indicates poorer performance in semantic judgment tasks as compared to healthy control groups, possibly affecting trials with both highly and weakly associated stimuli.

##### Quantitative Analyses

We carried out analyses from the 18 studies that reported mean accuracy scores as a function of age and semantic association. There was a small to moderate main effect of age (univariate ANOVA, *p* = 0.001, η_p_^2^ = 0.18), see Table 1, indicating lower accuracy scores in older as compared to younger participants (older: mean = 90.52%, *SEM* = 1.73; younger: mean = 92.93%, *SEM* = 1.47). There were no significant main effects of semantic association (*p* = 0.28, η_p_^2^ = 0.02) or of the interaction between age and semantic association (*p* = 0.22, η_p_^2^ = 0.03).

For patients with Alzheimer’s disease, accuracy was reported as a function of group and semantic association in only six studies. Therefore, we did not perform statistical analyses for accuracy.

##### Qualitative Review

**Figure 2:**
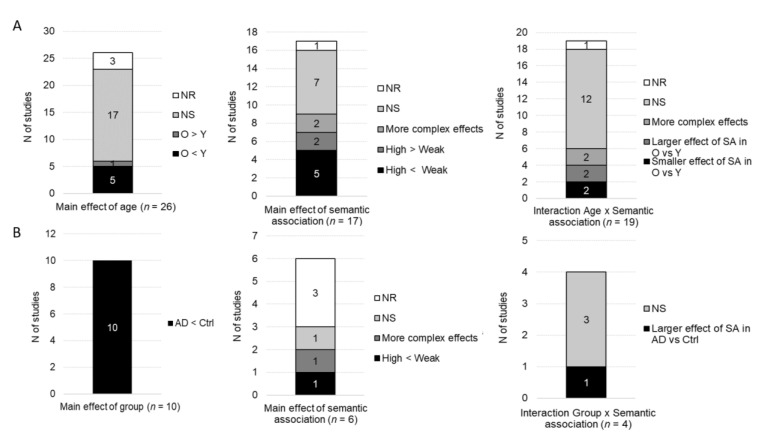
Qualitative results for behavioral accuracy: main effects of age/group, semantic association and the interaction group x semantic association. (**A**) Studies including older and younger groups of participants. (**B**) Studies including Alzheimer’s disease (AD) participants and control groups. N of studies: Number of studies; O: Older; Y: Younger; AD: Alzheimer’s disease; Ctrl: Control group(s); NS: No significant effect; NR: Statistical significance not reported; SA: Semantic association.

Overall, accuracy was measured as a function of age group in 26 studies in which participants performed a semantic judgment task, see Figure 2. From them, 17 studies found no main effect of age, six studies reported a main effect of age (five found lower accuracy for older than younger participants, whereas one found greater accuracy in older than younger groups), two studies did not report the significance of this effect, and one study did not report statistical comparisons for accuracy. Seventeen studies (without an AD group) investigated the main effect of semantic association. Nine of them found a main effect of semantic association (five found higher accuracy for highly than weakly associated stimuli, two found higher accuracy for weakly than highly associated stimuli, and two found more complex interactions involving more than two conditions), seven found no main effect of semantic association and one did not report the significance of this effect. Nineteen studies investigated the interaction between age and semantic association. Twelve of them found no interaction, six found a significant interaction, and one did not report the significance of this interaction. From the six studies that found significant interactions, two found a smaller effect of semantic association in older than young participants (with a numerical advantage for weakly vs. highly associated stimuli in the young group), two found a larger effect of semantic association in older than young participants (with a numerical advantage for highly vs. weakly associated stimuli in the older group) and two found more complex interactions involving more than two conditions.

All studies (*n* = 10) that compared accuracy in semantic judgment tasks between AD participants and control groups found a significant main effect of group with lower accuracy in AD participants. From these studies, six included a factor of semantic association in the analyses. Two of them found a main effect of semantic association (one found greater accuracy for weakly vs. highly associated stimuli and one found more complex interactions involving more than two conditions), one found no main effect of semantic association and three did not report whether the main effect of semantic association was significant or not. Four studies analyzed accuracy with factors of group and semantic association in the same statistical model. From them, only one found a significant interaction with a greater effect of congruence in AD participants as compared to the control groups, induced by a greater impairment for incongruent than congruent stimuli. The three other studies found no significant interaction.

#### 3.4.2. Behavioral Response Times

Both the quantitative results and qualitative review indicate longer RTs in older adults as compared to younger participants, regardless of the semantic association between stimuli. For AD patients, the qualitative review indicates that the AD group shows longer RTs in semantic judgment tasks and possibly a greater effect of semantic association as compared to healthy elderly participants.

##### Quantitative Analyses

We conducted quantitative analyses from the 12 studies that reported RTs as a function of age and semantic association using semantic judgment tasks. There was a large main effect of age (univariate ANOVA, *p* < 0.0001, η_p_^2^ = 0.44) and a moderate to large main effect of semantic association (*p* < 0.001, η_p_^2^ = 0.32), see Table 1. RTs were longer in older than younger participants (older: mean = 952.44 ms, *SEM* = 67.80; younger: mean = 840.25 ms, *SEM* = 75.38). RTs were shorter for highly than weakly associated stimuli (high: mean = 857.63 ms, *SEM* = 68.48; weak: mean = 935.06 ms, *SEM* = 75.71). The interaction between age and semantic association was not significant (*p* = 0.57, η_p_^2^ = 0.01).

RTs were available as a function of group and semantic association in only three studies involving an AD group and using semantic judgment tasks. Thus, we did not conduct statistical analyses.

##### Qualitative Review

**Figure 3:**
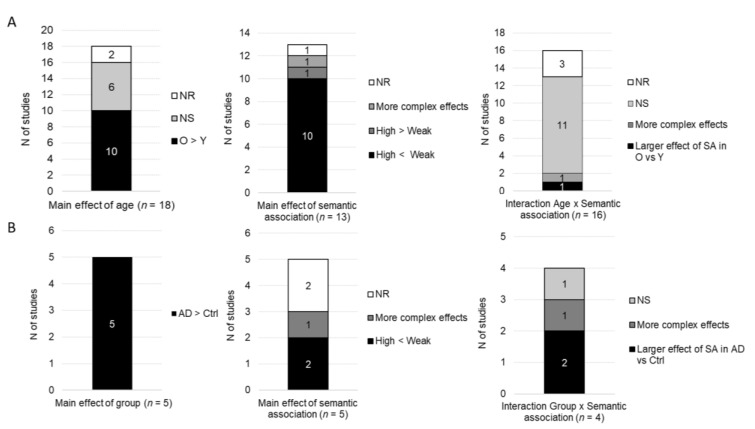
Qualitative results for behavioral response times: main effects of age/group, semantic association and the interaction group x semantic association. (**A**) Studies including older and younger groups of participants. (**B**) Studies including AD participants and control groups. *N* of studies: Number of studies; O: Older; Y: Younger; AD: Alzheimer’s disease; Ctrl: Control group(s); NS: No significant effect; NR: Statistical significance not reported; SA: Semantic association.

Overall, RTs were measured as a function of age group in 18 studies using semantic judgment tasks, see Figure 3. Ten of them found main effects of age, indicating longer RTs in older as compared to younger adults, six found no significant main effect of age, and two did not report the significance of this effect. Thirteen studies (without an AD group) analyzed RTs as a function of semantic association. Twelve found a main effect of semantic association (10 reported faster RTs for highly than weakly associated stimuli, one reported faster RTs for weakly than highly associated stimuli and one reported more complex interactions involving more than two experimental conditions) and one study did not report the significance of the effect of semantic association. Factors of age and semantic association were included in the same statistical model in 16 studies. Eleven of them found no significant interaction, two reported significant interactions, and three did not report the significance of the interaction. The two studies that found significant interactions indicate a greater effect of semantic association in older than young participants (i.e., greater RT difference between highly and weakly associated conditions; *n* = 1) and more complex interactions involving more than two experimental conditions (*n* = 1). From the 10 studies that we considered as nonsignificant, one found a greater effect of semantic association in older than young participants, but this difference was no longer significant when the overall longer RTs of the older group were taken into account.

RTs were analyzed in five studies with AD patients performing semantic judgment tasks. All found longer RTs in the AD group as compared to healthy control groups. These five studies also included a factor of semantic association in the analyses. Three found a main effect of semantic association (two reported faster RTs for highly than weakly associated stimuli and one reported more complex interactions involving more than two conditions) and two studies did not report the significance of the main effect of semantic association. The significance of the interaction between factors of group and semantic association was reported in four studies: two found a greater effect of semantic association in AD patients as compared to healthy control groups, one found that the effect of semantic association in AD participants was more similar to that of young adults than age-matched controls and one found no significant interaction.

#### 3.4.3. N400 Amplitude and N400 Effect Amplitude

The quantitative results and qualitative review show that the amplitude of the N400 effect is smaller in the elderly, as compared to young adults, especially due to smaller (i.e., less negative) N400 amplitudes when processing weakly associated stimuli. The qualitative review of studies with AD participants indicate that the N400 effect is smaller in the AD group as compared to healthy participants, but we cannot determine if this is due to N400 amplitude differences specifically for highly or weakly associated stimuli, or both.

##### Quantitative Analyses

**Figure 4:**
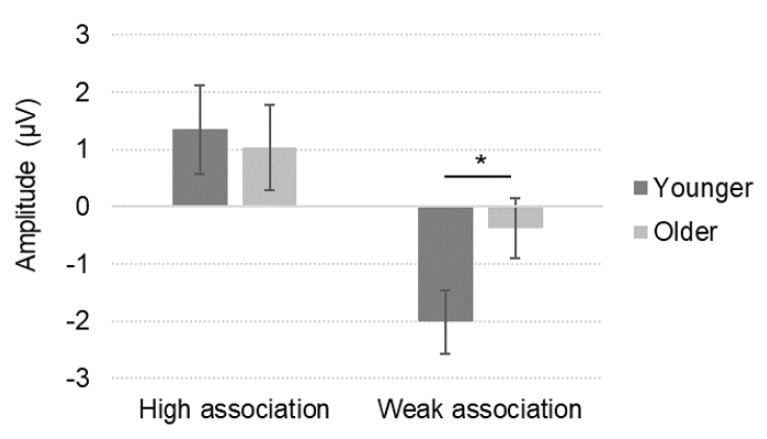
Mean N400 amplitude (± *SEM*) as a function of age group and semantic association in the reviewed studies (*n* = 21). * *p* < 0.05.

For the N400 amplitude, we conducted quantitative analyses from the 21 studies that reported mean N400 amplitude values as a function of age group and semantic association. There was a small main effect of age (univariate ANOVA; *p* = 0.042, η_p_^2^ = 0.07) and a large main effect of semantic association (*p* < 0.0001, η_p_^2^ = 0.52), see Table 1. Older participants had smaller N400 amplitudes than younger participants (older: mean = 0.33 μV, *SEM* = 0.39; younger: mean = −0.33 μV, *SEM* = 0.60). Highly associated stimuli elicited smaller N400 amplitudes as compared to weakly associated stimuli (high: mean = 1.19 μV, *SEM* = 0.47; weak: mean = −1.19 μV, *SEM* = 0.47). There was a small to moderate effect of the interaction between age and semantic association (*p* = 0.001, η_p_^2^ = 0.16). Test of simple effects indicated that weakly associated stimuli elicited a smaller N400 amplitude in older than younger adults. Further, highly associated stimuli induced a significantly smaller N400 amplitude than weakly associated stimuli in the older and younger groups (Figure 4).

For the N400 effect amplitude, values could be extracted or calculated as a function of age group from 31 studies. There was a large main effect of age (univariate ANOVA; *p* < 0.0001, η_p_^2^ = 0.75), see Table 1. The N400 effect was smaller (i.e., the amplitude difference between highly and weakly associated stimuli was smaller) for older than younger participants (older: mean = −1.46 μV, *SEM* = 0.16; younger: mean = −3.27 μV, *SEM* = 0.32).

For studies with AD participants, the mean values of amplitude for the N400 and N400 effect could be extracted or calculated respectively in only five and nine studies. Consequently, we did not perform statistical analyses.

##### Qualitative Review

**Figure 5:**
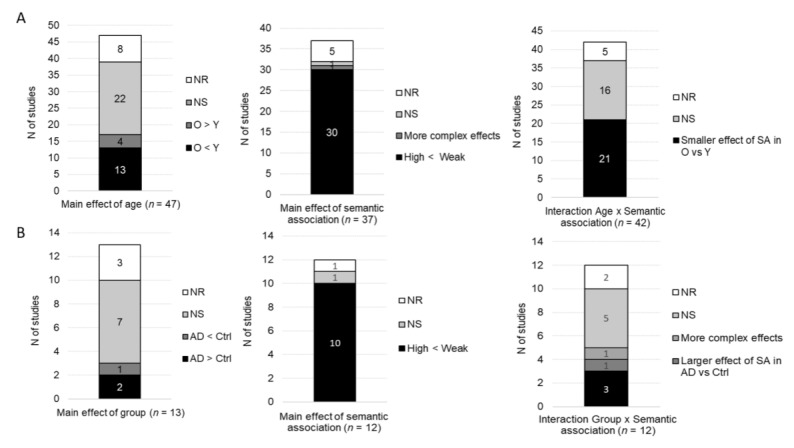
Qualitative results for the N400 amplitude: main effects of age/group, semantic association and the interaction group x semantic association. (**A**) Studies including older and younger groups of participants. (**B**) Studies including AD participants and control groups. N of studies: Number of studies; O: Older; Y: Younger; AD: Alzheimer’s disease; Ctrl: Control group(s); NS: No significant effect; NR: Statistical significance not reported; SA: Semantic association.

**Figure 6:**
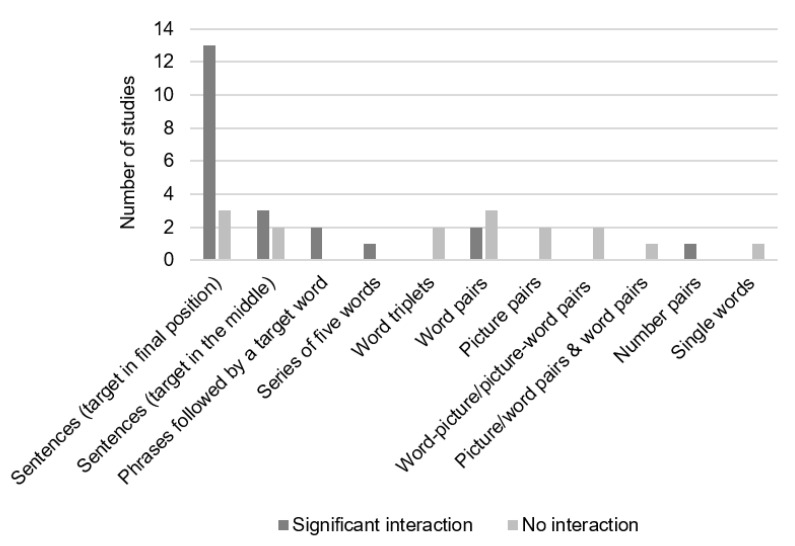
Number of studies that found a significant interaction between age and semantic association for the N400 amplitude as a function of stimulus type.

For the N400 amplitude, our literature search led to 47 studies that investigated the main effect of age on the N400 amplitude, see Figure 5. Twenty-two of them did not observe a significant age effect, 18 found a main effect of age and 7 did not fully report this effect. From the 18 studies that found a significant age effect, 13 observed smaller (i.e., less negative) N400 amplitude in older than younger adults, whereas four studies observed the reversed pattern and one study did not report the direction of the effect. Thirty-seven studies (without an AD group) included a factor of semantic association in the analyses. From them, 31 found a main effect of semantic association (30 reported larger N400 amplitudes for weakly than highly associated stimuli, one reported more complex interactions involving more than two experimental conditions), one found no main effect of semantic association and five did not report the significance of the main effect of semantic association. Forty-two studies performed analyses that included both factors of age group and semantic association. From them, 21 studies observed a significant interaction, whereas 16 reported nonsignificant interactions and 5 did not report the significance of the interaction. The 21 studies reporting significant interactions indicate that older as compared to younger participants showed smaller N400 amplitude differences between highly and weakly associated stimuli. Most of these studies measured the N400 amplitude in sentences with target words in the final position (see Figure 6 for the number of studies that found significant interactions between age and semantic association as a function of stimuli type).

For the N400 effect amplitude, 14 studies compared the amplitude between age groups. Nine of them found significant effect of age, indicating smaller N400 effects in older participants as a function of semantic association. Four studies did not find such significant age group differences and one did not report the significance of the effect.

Regarding AD patients, we found in the literature search that 13 studies investigated the effect of group on N400 amplitude. From these, only three reported significant main effects of group: two found greater N400 amplitudes (i.e., more negative) in the AD group as compared to the control groups, whereas one observed the contrary effect. Seven did not find a main effect of group and three did not report the significance of this effect. Twelve studies with AD patients investigated the effect of semantic association on N400 amplitude. Ten found a main effect of semantic association with greater N400 amplitudes for weakly than highly associated stimuli, one found no main effect of semantic association and one did not report the significance of this effect. Twelve studies investigated the interaction between group and semantic association. From these studies, five did not find a significant interaction, five found a significant interaction and two did not report the significance of the interaction. Among the studies that found a significant interaction, three found smaller N400 amplitude differences between highly and weakly associated stimuli in the AD group as compared to the healthy elderly group, one observed the reverse pattern and one study found greater N400 amplitudes for highly associated stimuli in AD participants as compared to elderly and young healthy participants, along with greater N400 amplitudes for weakly associated stimuli as compared to healthy elderly participants only.

Five studies compared the N400 effect between AD and controls groups. All found a significant main effect of group, indicating smaller N400 effect in the AD group as compared to the healthy groups.

#### 3.4.4. N400 Peak Latency and N400 Effect Peak Latency

Together, the quantitative analysis and qualitative review suggest that older participants tend to show similar N400 peak latencies but delayed N400 effect peak latencies as compared to younger participants. The qualitative review of AD studies suggests that the N400 effect latencies may be delayed in patients with AD.

##### Quantitative Analyses

Mean values of N400 peak latency were available as a function of age group and semantic association in only six studies. Therefore, we did not perform statistical analyses on this outcome.

N400 effect peak latency measures were available as a function of age group in 12 studies. There was a large main effect of age group (univariate ANOVA; *p* < 0.0001, η_p_^2^ = 0.82). The peak latency of the N400 effect occurred later in older participants as compared to younger participants (older: mean = 457 ms, *SEM* = 12; younger: mean = 404 ms, *SEM* = 11).

For AD participants, N400 latency values were not available as a function of group and semantic association in any of the reviewed studies. The peak latency of the N400 effect was available in only three studies, thus we did not conduct statistical analyses.

##### Qualitative Review

**Figure 7:**
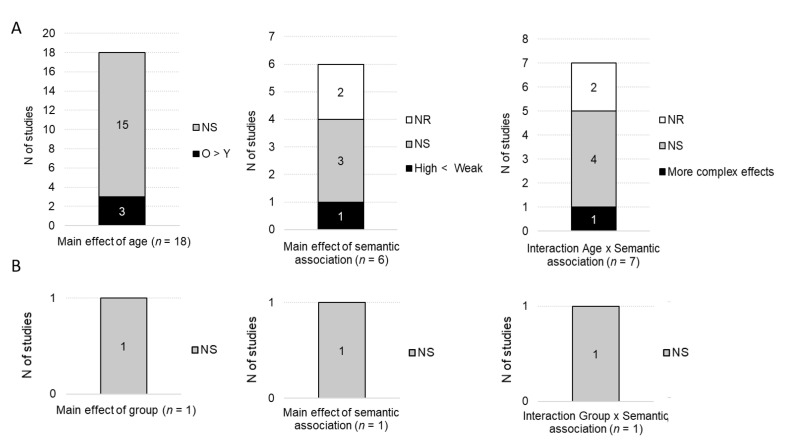
Qualitative results for the N400 peak latency: main effects of age/group, semantic association and the interaction group x semantic association. (**A**) Studies including older and younger groups of participants. (**B**) Studies including AD participants and control groups. *N* of studies: Number of studies; O: Older; Y: Younger; NS: No significant effect; NR: Statistical significance not reported.

For the N400 peak latency, 18 studies compared the N400 peak latencies between age groups, see Figure 7. Fifteen of them found no main effect of age, but three found a significant effect of age, with longer peak latencies in older as compared to younger participants. Six studies (without an AD group) included a factor of semantic association in the analyses. Three found no main effect of semantic association, one found a significant effect of semantic association with earlier peak latencies for highly than weakly associated stimuli and two did not report the significance of the effect of semantic association. Seven studies performed analyses with factors of age and semantic association in the same statistical model. Four of them found no significant interaction, one found a significant interaction involving more than two experimental conditions and two did not report whether the interaction was significant.

For the N400 effect, 13 studies investigated peak latency as a function of age. Eleven of them found longer latency in older as compared to younger participants, one study found no significant group effect and one study did not report the significance of this effect.

Only one study compared the N400 peak latency between AD and control groups. This study indicated that N400 latencies at Pz tended to be delayed in AD as compared to age-matched controls, although it did not reach significance (*p* < 0.06). No main effect of semantic association or the interaction between group and other variables were reported.

Four studies measured the peak latency of the N400 effect in AD participants. Two studies found longer N400 effect latencies in the AD group as compared to the control groups. One study found no differences between the AD group and age-matched controls but longer N400 effect latencies in AD participants as compared to young participants. One study did not report whether the main effect of group was significant.

## 4. Discussion

In this systematic review, we investigated differences in semantic processing in healthy aging and Alzheimer’s disease, as indicated by behavioral responses and N400 ERP measures. Furthermore, we examined whether these potential differences in semantic processing were modulated by the degree of semantic association of the stimuli.

The performance of healthy older adults was poorer than that of younger adults in semantic tasks. However, our qualitative review shows that most of the studies, considered individually, failed to find any differences in performance between young and elderly participants. Patients with AD performed more poorly than healthy elderly adults. There was no main effect of semantic association: highly and weakly associated stimuli showed a comparable performance, both in healthy (young and elderly adults) and impaired aging (elderly adults and patients with AD). Semantic association did not modulate the performance of any group of participants, either in healthy or pathological aging. Thus, performance in semantic tasks is impaired in AD but remains unclear in normal aging.

RTs were longer for elderly adults as compared to younger participants in semantic tasks. Patients with AD showed longer latencies than healthy elderly adults. There was a main effect of semantic association. Highly associated stimuli were responded to faster than weakly associated ones. The influence of semantic association on RTs was preserved in healthy aging. Conversely, our qualitative review showed that a few articles found a greater relatedness effect in patients with AD as compared to elderly adults. The RT differences found in healthy and pathological aging could be explained both in terms of semantic processing differences and general slowing, as will be discussed in later sections.

The N400 amplitude was influenced by healthy aging. Elderly adults showed overall smaller N400 amplitudes than young adults. Although this finding from the quantitative analysis was supported by several studies in the qualitative review, a large proportion of studies found no age differences. Elderly adults showed overall comparable N400 amplitudes to patients with AD. The results further indicate that semantic association affects N400 amplitudes: weakly associated stimuli elicited larger N400 amplitudes than highly associated ones. Critically, semantic association was modulated by the type of participant. The influence of semantic association was smaller in elderly than young adults and a small number of studies showed the same pattern in patients with AD compared to elderly adults. In conjunction with this, the N400 effect amplitude was smaller in older participants as compared to young adults. Based on our qualitative review, the N400 effect amplitude was also smaller in patients with AD than in healthy elderly adults. In healthy aging, this seems to be due to differences in the processing of weakly associated stimuli, while it remains unclear whether the processing of both highly and weakly associated stimuli differ from age-matched controls in AD.

The N400 peak latency was comparable in healthy and impaired aging, according to our qualitative review. Conversely, the N400 effect peak latencies appeared to be delayed in healthy aging and this may be also observed in pathological aging.

Thus, although it remains to clarify to what extent the behavioral performance of healthy elderly adults differs from that of young adults, there are clear differences in their electrophysiological responses of healthy elderly as compared to that of both young adults and AD patients. Therefore, it would be relevant to establish norms of healthy aging that could be eventually used for early AD detection. In that regard, behavioral performance, RTs and N400 effect amplitudes are the three outcomes for which healthy elderly and AD individuals differ. Consequently, tests norms for these three outcomes would be most relevant to distinguish healthy from pathological aging. Nonetheless, whether specific semantic variables (e.g., imageability, concreteness) have similar or distinct effects on healthy and pathological aging still needs to be investigated. This could allow researchers to determine if qualitative differences (e.g., deficits specific to AD), in addition to quantitative differences, may help identify pathological aging.

In the following sections, we discuss the interpretation of the results in more detail. For the sake of clarity, we present them separately for healthy and pathological aging.

### 4.1. Semantic Processing in Healthy Elderly and Young Adults

Results of the reviewed studies suggest age-related differences in semantic processing, as first hinted at by behavioral responses. The behavioral differences include an overall decrease in accuracy and increase in response times for healthy elderly adults. It is worth noting that the decrease in accuracy is small to moderate, as indicated by the partial eta-squared value. This could explain why most studies, at the individual level, did not find accuracy differences between young and older participants. Interestingly, in a meta-analysis of fMRI and PET studies, Hoffmann and Morcom [8] found that, on average, younger adults outperformed older adults on semantic tasks, even though this effect was small and often not significant in the reviewed studies. They also found with exploratory analyses that the reduction of activation in some regions involved in semantic processing, such as the left posterior middle temporal gyrus, only emerged in studies where older participants performed at a lower level than younger participants. In the current review, this kind of exploratory analysis was not possible since the number of studies which reported both accuracy and N400 amplitude values as a function of semantic association in a semantic judgment task is too small (*n* = 11) to separate them into subgroups.

Nonetheless, it is still possible that the behavioral differences highlighted by the current review result from age-related differences in EEG activity associated with semantic processing. Behavioral results could further reflect a slower access or even a less efficient access to the meaning of the items stored in semantic memory. Behavioral results from other studies also suggest age-related differences in semantic access or selection [34,35,36]. For instance, in a word association task when participants had to provide the first word that came to their mind for celebrity names, older participants made less precise associations than younger adults, which may reflect difficulties accessing detailed semantic knowledge [36]. In that regard, there is increasing evidence for the need to consider two aspects of semantic processing or semantic cognition, namely semantic knowledge and semantic control processes [1,3]. While semantic knowledge refers to the amount of knowledge acquired throughout the life course, semantic control processes allow us to retrieve and use this knowledge to perform cognitive tasks [1,3]. Hoffman [1] showed that elderly participants outperformed young participants in tasks assessing semantic knowledge (i.e., in a lexical decision task and a synonymy task with low-frequency words), whereas their accuracy was diminished compared to young participants in a feature association task, specifically in the condition when they had to ignore semantic associations irrelevant to the task. The inhibition of irrelevant semantic associations is underpinned by semantic control. There was also a link between accuracy in this condition and performance on a nonsemantic test of executive function (Wisconsin card-sorting test; [37]), for both elderly and young participants. Thus, it is plausible that differences in specific aspects of semantic cognition would occur with aging, potentially mediated by changes in executive functions more broadly, including inhibitory control.

The behavioral differences between elderly and young adults identified in this review seem to be similar for highly and weakly associated stimuli. This means that the influence of semantic association was comparable for both young and elderly adults. Consequently, an explanation of age differences in terms of more general processes would better explain these results. These more general processes may include potential differences in executive functions, a decrease in general processing speed [38] as well as a slowing of motor processes [39], which may likely contribute to the overall longer RTs in older adults. Since behavioral responses reflect multiple processes (at both the behavioral and neurophysiological levels), it is also possible that they were not sensitive enough to detect interactions between age and semantic association.

This review also indicates that age-related differences in semantic processing are especially evident in electrophysiological measures. So far, different interpretations have been proposed to explain the N400 differences found in older adults. One potential interpretation is the inhibition-deficit hypothesis [40], which postulates that older adults may fail to inhibit related items in working memory or have some difficulty suppressing irrelevant meanings. This interpretation could explain why the N400 amplitude reduction in older participants is particularly evident for weakly associated stimuli. Indeed, according to the integration view of the N400 component, the reduced N400 amplitude would reflect that the semantic features of the target stimuli have already been accessed, making them more easily (rightly or wrongly) integrated with previous information (see Kutas & Federmeier [15] for a review of the theories on the N400 component). This is expected for associated stimuli for which meaning integration is readily done. Nevertheless, this N400 amplitude reduction was also found for weakly associated stimuli in the elderly. This suggests that the semantic processing of highly and weakly associated stimuli is less differentiated in the elderly.

Another interpretation is linked to the amount of cumulated semantic knowledge (e.g., [1]). As semantic knowledge increases with age [6], and since synaptic plasticity mechanisms are associated with learning and memory formation [41,42], some connections within the semantic network might be strengthened with age, thus causing an easier integration of distant concepts for older adults, as reflected by a smaller N400 for weakly associated stimuli.

Finally, another interpretation relates to potential differences in lexical prediction mechanisms (e.g., [43]). Specifically, the lexical prediction (i.e., the anticipation of upcoming words) might be more important in younger than in older adults, which may reflect a larger N400 amplitude for weakly associated stimuli in younger participants. This is particularly depicted when studies use sentences instead of isolated words or pairs of words as stimuli. The previous words of a sentence may act as a context that helps lexical prediction for the target word in a sentence. Consequently, the later the target word in a sentence, the more a context is created for prediction. Accordingly, most studies reporting significant interactions between age and semantic association used sentences with target words in the final position. During a sentence comprehension task, Dave et al. [44] measured the effects of both prediction accuracy (i.e., words accurately predicted vs. not predicted by the participants) and contextual support related to semantic association (i.e., low vs. moderate cloze words, the former being incongruent) in older and younger adults. They found that the N400 effect was smaller in older participants for both prediction accuracy and contextual support, independently of each other. Furthermore, older and younger participants did not differ in the percentage of final words correctly predicted, for both moderate and low cloze words. Results from Dave et al. [44] suggest that older participants do predict upcoming words to a comparable degree to young adults. This strengthens the idea that differences in processes other than lexical prediction might contribute to the N400 effect reduction typically observed in older adults. In addition, some studies found reduced N400 effects in older participants using less constraining contexts (e.g., with word pairs; see for example Gunter et al. [24], Miyamoto et al. [10]) or even subliminal priming [19]. These suggest that these age-related differences would not be solely due to prediction mechanisms. In sum, factors such as differences in inhibition processes, the amount of semantic knowledge or predictive mechanisms may all contribute, at least in part, to the N400 differences observed in older adults.

Interestingly, and in light of the explanation of semantic differences in terms of other cognitive functions, such as semantic control/inhibition, a few studies investigated the associations between the N400 and behavioral outcomes using correlations or regressions. Some reported no significant associations between N400 measures and accuracy of semantic judgments [11] or inhibition [45]. Some authors found that the latency of the N400 effect of older participants was delayed with decreasing performance at a reading span task to assess working memory [25]. However, there was no association between reading span and the size of the N400 effect in this study, nor with N400 amplitude measures in two other studies [45,46]. Some studies also reported associations between N400 amplitude measures and verbal fluency (tasks that combined phonological and semantic fluency) or vocabulary [45,46]. Thus, there is a possibility that working memory, executive functions and/or lexical processing might explain some individual variability in the N400 ERP. However, causal links between these elements, as well as the potential impact of N400 differences on behavioral responses in semantic tasks, still need to be further investigated. In future studies, analyses at the level of individual items may help us determine whether the EEG differences observed in older adults are positive, negative or even have a negligible impact on behavior related to explicit semantic processing.

### 4.2. Semantic Processing in Individuals with Alzheimer’s Disease

The behavioral results of our systematic review indicate impaired performance and longer RTs in AD participants as compared to their age-matched counterparts when they make decisions about the meaning of stimuli. The performance of AD participants is diminished for semantic processing of both highly and weakly associated stimuli. In accordance with these results, previous behavioral studies reported impaired performance of AD patients in a variety of tasks assessing semantic knowledge [5,47,48], semantic control/inhibition [49], as well as more semantic errors in naming tasks [50]. However, we still need to consider that other cognitive and motor processes are susceptible to contribute to the decreased performance in semantic judgment tasks in AD patients. As it might be the case for healthy aging, longer RTs in AD patients may also be due, at least in part, to a general slowing of processing speed [51]. Furthermore, the potential greater relatedness effect observed in RTs of AD patients may be explained by these overall longer RTs. In order to examine this possibility, Schwartz et al. [18] performed a regression analysis using a total RT measure to predict the RT difference between unrelated and related trials. They found that the regression equations calculated from young participants were good predictors of the relatedness effect of healthy elderly participants but underestimated the relatedness effect of AD participants at the basic and subordinate levels (i.e., experimental conditions where they showed significant relatedness effects). This suggests that the extent of the slowing in RTs differed between semantic conditions. Thus, in this case, the interaction between groups and relatedness would be in favor of semantic processing differences between AD patients and healthy individuals, rather than differences in earlier/other processes such as motor planning, that would typically affect both types of semantic conditions.

The studies reviewed in this article are also in favor of differences in EEG activity associated with semantic processing in individuals with AD as compared to healthy elderly adults. In fact, semantic association seems to have less impact on N400 amplitudes in AD than in healthy elderly participants. Semantic processing may also be delayed in AD patients, as indicated by longer N400 effect latencies. These results may be explained either by deficits in semantic access (see the explanation in terms of different semantic control capacities in the previous section; [1,3]) or by a deterioration of the semantic representations themselves. Both semantic access and semantic deficits have been reported in AD patients. Notably, access deficits might be more prominent at the beginning of the disease, but a progressive loss of semantic knowledge appears to occur with increasing disease severity [4,52,53]. Relatedness and congruence effects in the N400 time-window have been mainly localized in left temporal regions (including the left anterior and medial temporal cortices; [54,55,56,57]), where AD patients show glucose hypometabolism [58], white matter microstructural abnormalities [59] and gray matter volume loss [60]. This supports the idea that reduced N400 effects may result from damage to the semantic network. Incidentally, in AD patients, behavioral performance at semantic memory tasks has been previously associated with several biomarkers in key regions of the semantic network, such as glucose metabolism and tau pathology in the left anterior temporal regions [61,62,63,64]. However, AD patients typically display deficits in multiple cognitive functions, including executive functions, attention and working memory [65]. Consequently, these deficits may also play a role in N400 differences. The N400 can be modulated by attention [66,67] and verbal working memory [68] in healthy participants. Thus, as for age-related N400 differences, multiple aspects of cognitive functions may contribute to the N400 differences observed in AD patients as compared to healthy elderly participants.

Some authors found associations between the N400 and neuropsychological measures. For instance, the N400 effect was positively correlated with scores to the MMSE, Wechsler Adult Intelligence Scale (WAIS; [69]) similarities subtest (semantic conceptual reasoning task) and explicit detection of incongruous sentences, when considering both AD and healthy elderly participants [70]. Performance at several neuropsychological tests also predicted fractional area latencies in the opposite (antonym) condition, as indicated by a multiple linear regression [17]. In addition, abnormal N400 topography has been associated with decreased cerebral blood flow in the left and right temporal lobes, as well as impaired performance in the MMSE, the Boston Naming Test [71] and animal and verb fluency tasks [72]. Finally, within group analyses in Ford et al. [73] revealed that the N400 effect of AD patients did not vary as a function of their capacity to name the pictures used as primed stimuli. Together, these results support, to some extent, the hypothesis that N400 differences in AD reflect semantic deficits, although it remains an associative link rather than a causal one.

Differences in the N400 ERP may either reflect a functional reorganization involving the semantic cognition network or compensatory mechanisms. The fact that semantic knowledge is preserved in healthy aging, while semantic deficits are observed in AD, supports the idea that these deficits do not simply reflect an accelerated aging process [7]. However, the current review suggests that performance in semantic judgment tasks, which require semantic control processes is diminished in older as compared to young adults [74]. This does not preclude the possibility of both quantitative and qualitative changes from healthy aging to AD, at least in terms of semantic processing.

### 4.3. Limitations and Perspectives

This systematic review contributes to establish a more comprehensive view of semantic processing in healthy aging, through mid- and late adulthood. In the reviewed studies, the mean age of participants in the groups of elderly varied between 50 and 75 years of age. However, this review focused on between-group differences rather than longitudinal changes within a same individual or group. This prevents us from determining whether there are linear relationships between age and outcomes on semantic tasks. Kutas and Iragui [20] measured N400 changes across six decades, with 72 participants (12 participants per decade) aged from 20 to 80 years old. They reported a linear decrease in the amplitude of the N400 (0.05–0.09 μV/year) with a linear increase in N400 peak latencies with age (1.5–2.1 ms/year). However, since studies also indicated curvilinear patterns for verbal knowledge [75,76], we cannot exclude that the same pattern may apply to the N400. The study of time course changes is rare in the literature since most studies are cross-sectional, thus testing for differences between groups (e.g., elderly vs. young adults; healthy vs. AD), instead of longitudinal, that is testing for changes within a given individual at various time points. The behavioral performance and the electrophysiological signature of semantic processing may have differential temporal courses of changes. An individual might display some decline in the electrophysiological responses such as smaller amplitude or longer peak latency signal that might not *yet* be captured behaviorally by existing semantic tasks. Further, we may find that differences in the N400 effect are not linked to behavioral responses, which could represent potential decline that we can identify in the electrophysiological responses prior to behavioral decline. This may contribute to detect decline before it becomes a full AD picture. Therefore, we propose for future work to study behavioral and electrophysiological responses during semantic processing with a longitudinal design, which will contribute to depicting their time course and clinical relevance.

There are also some methodological limits in the reviewed studies here that have to be taken into account. First, a better description of different key elements would be advisable, such as information regarding EEG recording and preprocessing (e.g., impedances, number of remaining trials per group/condition) and the justification for the choice of electrode location and time-windows. All results should also be reported regardless of their statistical significance. In case of multiple testing (e.g., for each individual electrodes or multiple time-windows), a correction procedure for multiple comparisons should be used in order to avoid type 1 errors. Moreover, insufficient reporting in EEG studies in general, including the lack of information regarding sample size calculation, has been previously raised and complicates the replicability of results [77,78]. At a more technical level, it is recommended that studies measuring the N400 use a sampling rate of 250 Hz [79] and a high-pass filter ≤0.1 Hz in order to avoid artifactual P2 effects and maximize statistical power [80]. In some of the reviewed studies, sampling rate and high-pass filter were respectively below and above these values.

Another important issue is that stimuli were not always matched for psycholinguistic variables known to have an influence on the N400, such as lexical frequency [57], neighbourhood size [81], imageability [82] and concreteness [83]. Furthermore, it is well known that reading processes are modulated by word length [84]. Thus, words from different semantic conditions should be comparable in terms of these variables to ensure that the differences between groups are specific to the semantic variable of interest. For instance, Huang et al. [85] found a reduced N400 effect for concreteness in elderly participants, while most studies reviewed here did not control for this semantic variable. One way to improve matching of target words between conditions is that all of them appear once in each semantic condition, as elegantly done in some studies (e.g., [12,46,86]).

Finally, this systematic review studied the effects of semantic association (sometimes called semantic priming) in general, but it does not allow to distinguish the effects of predictability and congruence per se. We acknowledge that these effects are not identical, since they show different topographies in late ERP positivities [87] and are associated with activity in different brain regions, as reported by fMRI studies [88]. However, target stimuli can be both highly predictable and congruent or unpredictable and incongruent. Many of the reviewed studies did not manipulate these variables within the same design, thus we could not specifically separate them. Of note, these continuous variables may be seen as two overlapping dimensions of a larger semantic continuum. We could find at one end of this continuum congruent and highly predictable stimuli (underlying strong semantic association) and completely incongruent and unpredictable stimuli at the other end (underlying the total absence of association), with congruent but unpredictable stimuli in the middle. Even within studies investigating relatedness effects, there may be a wide range of association strength (e.g., related stimuli could be either highly or moderately related). Thus, this review does not capture all of these subtleties and their potential moderating effects on semantic processing in aging and AD. However, since more attempts have recently been made to manipulate such variables separately, we should be able in the future to better understand their specific contributions to semantic processing.

## 5. Conclusions

This systematic review reinforces the idea that differences in semantic processing are observed in healthy elderly adults relative to both young and AD patients. The differences between elderly and young healthy adults are especially evident in the electrophysiological responses and could reflect either a functional reorganization within the semantic cognition network or compensatory mechanisms. The influence of semantic association on the N400 ERP measures is smaller in elderly adults as compared to young adults and even smaller in AD patients as compared to healthy elderly adults. These differences in semantic processing possibly underlie differences in semantic access, in the richness of semantic representations and/or in other cognitive processes such as semantic control or, more broadly, executive functions. Since AD patients differ from healthy elderly participants in terms of both behavioral and electrophysiological responses, test norms related to these correlates of semantic processing could be useful for the purposes of early AD detection.

## Figures and Tables

**Table 1:** Results of univariate ANOVAs and tests of simple effects for comparisons between older and younger healthy adults.

Outcome	Factor	*df*	*F* Value	*p* Value	η_p_^2^
Accuracy	Age	1/51	11.435	0.001 *	0.183
	Semantic association	1/51	1.192	0.280	0.023
	Age × Semantic Association	1/51	1.523	0.223	0.029
	Study	17/51	19.647	0.000 **	0.868
RTs	Age	1/33	25.934	0.000 **	0.440
	Semantic association	1/33	15.505	0.000 **	0.320
	Age × Semantic Association	1/33	0.323	0.574	0.010
	Study	11/33	78.584	0.000 **	0.963
N400 amplitude	Age	1/60	4.338	0.042 *	0.067
	Semantic association	1/60	64.889	0.000 **	0.520
	Age × Semantic Association	1/60	11.128	0.001 *	0.156
	High association: O vs. Y	1/20	0.764	0.392	0.037
	Weak association: O vs. Y	1/20	13.272	0.002 *	0.399
	Older: High vs. Weak	1/20	52.716	0.000 **	0.725
	Younger: High vs. Weak	1/20	77.119	0.000 **	0.794
	Study	20/60	16.497	0.000 **	0.846
N400 effect amplitude	Age	1/30	89.605	0.000 **	0.749
	Study	30/30	5.287	0.000 **	0.841
N400 effect peak latency	Age	1/11	51.393	0.000 **	0.824
	Study	11/11	12.476	0.000 **	0.926

Note: *df:* degrees of freedom; RTs: Response times; O: Older; Y: Younger. * *p* < 0.05; ** *p* < 0.001.

## Supplementary Materials

The following are available online at https://www.mdpi.com/2076-3425/10/11/770/s1, Table S1: PRISMA checklist, Table S2: Detailed search terms.

## Appendix A

**Table A1:** Main characteristics of participants, materials and procedures and results of each study including older and younger groups of participants.

Reference	N of Subjects (O; Y)	Mean Age (O; Y)	Female % (O; Y)	Task ^1^	Stimuli ^2^	N of Electrodes ^3^	N400 Time-Window (ms)	Main Results ^4^	
								N400	Behavior ^5^
Harbin et al., 1984 [89]	12; 12	71; 21	0; 0	Semantic judgment	Series of 5 words	3 (Fz, Cz, Pz)	300–600	- N400 amplitude: Age: n/a, match < mismatch, Age × Target (target effect in Y only)	- Accuracy: n/a - RTs: Age: n/a, match > mismatch, Age × Target: n/a, Age × Task × Target
Gunter et al., 1992 [90]	24; 24	56; 22	n/a; n/a	Sentence reading	Sentences	8 (Fz, Cz, Pz, Oz, Tl, Tr, Ml, Mr)	30 ms time-windows from 10 to 990 ms	- N400 amplitude: Age: n/a, congruent < incongruent between 340 and 550 ms, Age × Congruence (smaller congruence effect in O vs. Y from 310 to 460 ms after target onset)	—
Hamberger, & Friedman, 1992 [91]	18; 18	70; 25	100; 100	Semantic judgment	Single words	13	O: 250–500; Y: 300–450	- N400 amplitude: O = Y, primed < unprimed category, Age × Priming: n/a	- Accuracy: n/a - RTs: O = Y, primed < unprimed category, Age × Priming: n.s.
Gunter et al., 1995 [92]	24; 24	58; 21	42; 50	Sentence reading	Sentences	8 (Fz, Cz, Pz, Oz, Tl, Tr, Ml, Mr)	Mean amplitude: 30 ms time-windows from 10 to 750 ms, Peak amplitude/ latency: 400–650	- N400 amplitude: O = Y, congruent = incongruent, Age × Congruence: n.s. - N400 peak latency (incongruent trials): O > Y	—
Gunter et al., 1996 [93]	24; 24	58; 21	42; 50	Sentence reading	Sentences with flanker word to be ignored, beneath the target	7 (Fz, Cz, Pz, Oz, Tl, Tr, Mr)	Mean amplitude: 30 ms time-windows from 10 to 750 ms	- N400 amplitude: O < Y between 340 and 730 ms, congruent < incongruent between 310 and 510 ms, Age X Congruence (smaller congruence effect in O vs. Y from 340 to 400 ms after target onset)	—
Gunter et al., 1998 [24]	20; 20	57; 21	50; 50	Word reading	Word pairs	7 (Fz, Cz, Pz, Oz, Tl, Tr, Mr)	Mean amplitude: 30 ms time-windows from 0 to 750 ms, peak amplitude/latency: 300–600	- N400 amplitude (for targets): O = Y, associated < nonassociated between 290 and 740 ms, Age × Congruence (smaller association effect in O vs. Y from 260 to 560 ms after target onset, driven by smaller N400 for nonassociated targets in O vs. Y), Age × Association X Electrode between 320 and 540 ms - N400 peak latency: O = Y, Congruence: n/a, Age × Congruence: n/a	—
Kutas & Iragui, 1998 [20]	12; 12	74; 24	50; 42	Semantic judgment	Phrases followed by a target word	13	200–600	- N400 amplitude: Age: n/a, congruent < incongruent, Age × Congruence (smaller congruence effect in O between 200 and 400 ms) - N400 effect amplitude: O < Y - N400 effect onset latency: O > Y	n/a
Miyamoto et al., 1998 [10]	12; 12	66; 24	0; 0	Semantic judgment	Word pairs	3 (Fz, Cz, Pz)	O: 276–326; Y: 226–276 (match) & 276–326 (mismatch)	- N400 amplitude: O = Y, Matching: n/a, Age × Matching: n/a, Age × Matching × Site (smaller matching effect in O vs. Y at Fz, driven by smaller N400 for mismatch trials in O vs. Y)	- Accuracy: O < Y, match = mismatch, Age × Matching: n.s. - RTs: O > Y, match < mismatch, Age × Matching: n.s.
Cameli & Phillips, 2000 [94]	20; 20	72; 23	60; 60	Sentence & word reading	Sentences & word pairs	15	300–600	- N400 amplitude: Age: n/a, Relatedness: n/a, Age × Relatedness (smaller relatedness effect in O vs. Y for both sentences and word pairs) - N400 peak latency: O = Y, Relatedness: n/a, Age × Relatedness: n/a	—
Chaby et al., 2001 [9]	12; 14	53; 25	50; 50	Semantic judgment	Picture pairs	30	350–450	- N400 amplitude: O > Y, congruent < incongruent, Age × Congruence: n.s. - N400 peak latency: O > Y, congruent < incongruent, Age × Congruence: n.s.	- Accuracy: O < Y, congruent > incongruent, Age × Congruence (congruent > incongruent for O only) - RTs: O = Y, congruent < incongruent, Age × Congruence: n.s.
Bonnaud et al., 2002 [95]	10; 10	66; 24	n/a; n/a	Semantic judgment	Word pairs	3 (Fz, Cz, Pz)	300–600	- N400 amplitude: O < Y, Nonmetaphorical semantic link < metaphorical semantic link = no semantic or phonological link, Age × Condition: n.s. - N400 peak latency: O > Y, Condition: n.s., Age × Condition: n.s.	- Accuracy: O > Y, Metaphorical semantic link < Nonmetaphorical semantic link & No semantic or phonological link, Age × Condition (O = Y for nonmetaphorical semantic link and trials without semantic or phonological link, O > Y for metaphorical semantic link) - RTs: O = Y, Nonmetaphorical semantic link < metaphorical semantic link = No semantic or phonological link, Age × Condition (O = Y for metaphorical and nonmetaphorical semantic link, O>Y for trials without semantic or phonological link)
Federmeier et al., 2002 [46]	24; 21	68; 20	50; 52	Sentence listening	Sentence pairs (context sentence, followed by the target-containing sentence)	26	300–500	- N400 amplitude: O < Y, context: n/a, Age × Context: n/a - N400 peak latency: O = Y, context: n/a, Age × Context: n/a	—
Federmeier et al., 2003 [13]	20; 20	72; 22	50; 65	Semantic judgment	Sentences (targets in multiple positions)	15	200–500 (for sentence final words)	- N400 amplitude (sentence final words): O = Y, congruent > anomalous, Age × Congruence (smaller congruence effect in O vs. Y for sentence final words), Age × Congruence × Electrode (smaller congruence effect in O vs. Y especially at medial sites) - N400 peak latency (anomalous trials): O = Y	- Accuracy: O = Y, congruent > anomalous, Age × Congruence: n.s. - RTs: n/a
Phillips & Lesperance, 2003 [96]	Exp 1: 20; 19 Exp 2: 13; 10	Exp 1: 77; 23, Exp 2: 72; 24	Exp 1: 70; 74, Exp 2: 46; 50	Sentence reading with distractor words (exp 1) or without distractors (exp 2)	Sentences followed by a target word	17	Exp 1: 300–600; Exp 2: Y: 400–450; O: 550–600	- N400 amplitude (Exp 1): O < Y, Relatedness: n/a, Age × Relatedness: n/a - N400 peak latency (Exp 1): O = Y, sentence-related = distractor-related = unrelated, Age × Relatedness: n/a - N400 effect amplitude (Exp 2): O = Y	—
Fabre & Lemaire, 2005 [19]	11; 11	69; 24	46; 55	Semantic (parity) judgment with semantic masked priming	Number pairs (arabic numerals or number words)	32	O:495–550; Y:370–485 & O:515–535, Y:410–430	- N400 amplitude: Age: n/a, congruent < incongruent, Age × Congruence (smaller congruence effect in O vs. Y at parietal sites)	- Accuracy: O = Y, congruent = incongruent, Age × congruence: n.s. - RTs: O > Y, congruent < incongruent, Age × congruence: n.s.
Federmeier & Kutas, 2005 [25]	20; 20	67; 20	50; 50	Sentence reading	Sentences	26	300–500	- N400 amplitude: O = Y, weakly > highly constrained contexts, Age × Constraint (smaller constraint effect in O vs. Y, driven by larger N400 for highly predictable targets in O vs. Y over central and posterior electrode sites) - N400 peak latency (weakly constrained contexts): O = Y - N400 effect peak latency: O > Y	—
Nessler et al., 2006 [97]	16; 16	72; 23	44; 69	Semantic judgment	Picture-word pairs & word pairs	62	400–800	- N400 amplitude: O < Y, low < high semantic selection demands, Age × Semantic selection demands: n/a	- Accuracy: O = Y, match = mismatch (low selection condition), Age × Matching (low selection condition): n.s. - RTs: O = Y, Matching: n/a, low < high semantic selection demands, Age × Matching: n.s., Age × Semantic selection demands: n.s.
Faustmann et al., 2007 [98]	12; 13	75; 56	58; 62	Semantic judgment	Sentences (targets in the middle)	30	250–500	- N400 amplitude: Age: n/a, congruent < incongruent, Age × Congruence (smaller congruence effect in O vs. Y) - N400 peak latency (incongruent trials): O = Y - N400 effect peak latency: O = Y	- Accuracy: Age: n/a, congruent = incongruent, Age × Congruence: n.s. - RTs: n/a
Federmeier et al., 2010 [99]	Exp 1: 20; 16	68; 20	55; 56	Semantic judgment	Phrasal cues followed by a target word	26	O:350–550; Y:300–500	- N400 amplitude: O = Y, expected < incongruent, Age × Congruence (but significant effects of congruence in the antonym and category conditions for both O and Y groups) - N400 effect peak latency: O > Y, Antonym > Category, Age × Condition: n.s.	- Accuracy: Age: n/a - RTs: n/a
Kawohl et al., 2010 [100]	10; 10	60; 27	n/a; n/a	Sentence reading	Sentences	20	300–500	- N400 amplitude: Age: n/a, congruent < incongruent, Age × Congruence (congruence effect in Y only)	—
Kousaie & Phillips, 2011 [101]	15; 16	72; 24	60; 56	Lexical decision with semantic priming	Word triplets	29	50 ms time-windows from 300 to 700 ms after target onset	- N400 amplitude: O = Y, related < unrelated, Age × Relatedness: n.s., Age × Relatedness × Time × Language	—
Lee & Federmeier, 2011; 2009 [102,103]	24; 24	68; 20	50; 50	Sentence reading	Sentences	26	250–500	- N400 amplitude: O = Y, congruent < syntactic prose sentences with no coherent semantics, Age × Context (smaller context effect in O vs. Y)	—
Grieder et al., 2012 [104]	15; 14	69; 27	73; 43	Lexical decision with semantic priming	Word pairs	32	Variable	- Microstate cluster analysis: Delayed N400-LPC microstate in O vs. Y and unrelated vs. related trials, Age × Relatedness: n.s. - Global field power: O = Y, related > unrelated, concrete > abstract, Age × Relatedness: n.s., Age × Concreteness: n.s.	—
Huang et al., 2012 [85]; Huang et al., 2010 [105]	20; 32	65; 20	60; 50	Semantic judgment	Adjective-noun pairs	26	300–500	- N400 effect amplitude: O = Y for the congruence effect, O < Y for the concreteness effect - N400 effect peak latency (congruence): O > Y	- Accuracy: O = Y, Congruence: n/a, Age × Congruence: n/a - RTs: n/a
Lee & Federmeier, 2012 [106]	24; 16	66; 19	50; 50	Sentence reading	Sentences (targets in the middle)	26	300–600	- N400 amplitude: O < Y, plausible < implausible, Age × Plausibility (smaller plausibility effect in O vs. Y for sentences with preceding ambiguity, but not for sentences without preceding ambiguity)	—
Wlotko & Federmeier, 2012 [43]	20; 16	68; 20	45; 50	Sentence reading	Sentences	26	O: 325–525; Y: 280–480	- N400 amplitude: Age: n/a, Cloze probability: n/a, Age × Cloze probability (smaller variation as a function of cloze probability in O vs. Y in weakly constraining sentences only) - N400 effect amplitude: O < Y - N400 effect peak latency: O > Y	—
Wlotko et al., 2012 [21]	24; 24	72; n/a	50; n/a	Sentence reading	Sentences	26	Mean amplitude: O: 325–525; Y: 275–475, peak latency: 300–500	- N400 effect amplitude (expectancy & constraint for expected endings): O < Y - N400 effect peak latency (expectancy and constraint for expected endings): O > Y	—
Davis et al., 2013 [107]	20; 20	50; 22	100; 100	Semantic judgment during continuous competing speech	Word pairs	30	300–600	- N400 amplitude: O < Y, Relatedness: n/a, Age × Relatedness: n/a - N400 peak latency: O = Y, Relatedness: n/a, Age × Relatedness: n/a	- Accuracy: O = Y, different> same semantic category, Age × Relatedness: n.s. - RTs: O = Y, same<different semantic category, Age × Relatedness: n.s.
Molnár et al., 2013 [108]	14; 15	66; 21	79; 47	Semantic judgment	Single words	33	250–500	- N400 amplitude: Age: n/a, target (words matching a target valence category) < nontarget category, Age × Target: n/a	- Accuracy: Age: n/a, Target: n/a, Age × Target: n/a, Age × Target × Valence (for negative words, higher accuracy for nontarget vs. target category in Y only) - RTs: n/a
Wilkinson et al., 2013 [86]	18; 18	70; 21	83; 83	Semantic judgment	Prime word and superimposed but to-be-ignored picture followed by a target word	20	Mean amplitude: variable (200 ms time window centered around peak latency for each age group), peak latency: 300–800	- N400 amplitude: O = Y, related < unrelated, Age × Condition: n.s., Age × Condition × Interstimulus interval (smaller N400 for trials with related target and distractors vs. trials with related targets and unrelated distractors in Y only, in the 1000 ms ISI condition) - N400 peak latency: O = Y, Condition: n/a, Age × Condition: n/a	- Accuracy: O = Y, trials with related target and distractor and unrelated target and distractor > unrelated target and related distractor and related target and unrelated distractor, Age × Condition (trials with related target and distractor & unrelated target and distractor in O > related target and unrelated distractor in Y) - RTs: O > Y, related target and distractor < related target and unrelated distractor < unrelated target and distractor < unrelated target and related distractor, Age × Condition: n.s.
Mehta & Jerger, 2014 [109]	10; 11	Range: 60–0; 20–0	60; 46	Semantic judgment	Word triplets	30	250–750	- N400 amplitude: Age: yes (no post-hoc for O vs. Y), related < unrelated, Age × Relatedness: n.s.	—
Zhou et al., 2015 [110]	15; 15	68; 23	47; 40	Semantic judgment	Single words	64	300–550	- N400 amplitude: O = Y, congruent < incongruent, Age × Congruence: n.s.	- Accuracy: O = Y, congruent = incongruent, Age × Congruence: n.s. - RTs: O > Y, congruent < incongruent, Age × Congruence: n.s.
Chang et al., 2016 [111]	7; 16	58; 23	29; 63	Sentence reading	Sentences	64	1st analysis: 250–500, 2nd analysis: 50 ms time-windows from 250 to 700 ms	- N400 amplitude: 1st analysis: Age: n/a, low > high predictability, Group × Predictability, Group × Predictability × Anteriority (smaller predictability effect in O vs. Y at frontocentral, central and posterior sites); 2nd analysis (post-hoc tests): Predictability effects from 300 to 550 ms in O vs. from 250 to 450 ms in Y	—
Ghosh Hajra et al., 2016 [112]	6; 6	Range: 50-85; 20-30	67; 50	Word-pair listening	Word pairs	3 (Fz, Cz, Pz)	n/a	- N400 amplitude (incongruent trials): O = Y - N400 peak latency (incongruent trials): O = Y	—
Khachatryan et al., 2017 [113]	12; 20	53; 21	83; 45	Semantic judgment	Sentences	32	1st analysis: O: 300–500; Y: 274.2–500, 2nd analysis: 50 ms time-windows from 250 to 600 ms	- N400 amplitude: 1rst analysis: O > Y, congruent < incongruent, Age × Congruence (smaller congruence effect in O vs. Y); 2nd analysis: Age: n/a, Age × Sentence: n/a, Age × Sentence × Time-windows: n.s.	- Accuracy: O = Y, congruent > incongruent, Age × Congruence: n/a - RTs: n/a
Payne & Federmeier, 2017 [114]; Stites et al., 2017 [115]	23; 24	68; 21	67; 21	Sentence reading	Sentences (targets in the middle)	26	Parafoveal N400 window: 400–600, foveal N400 window: 850–1050	- N400 effect amplitude: Age: n/a, Age × Foveality (comparable congruence effect in O vs. Y when the target stimuli first appeared in parafoveal vision, but larger congruence effect in O vs. Y when the target stimuli then moved at central fixation) - N400 effect fractional area latency: O > Y	—
Weißbecker-Klaus et al., 2017 [116]	20; 21	57; 27	55; 43	Semantic judgment with concurrent flanker task	Word pairs	64	n/a	- N400 amplitude: O = Y, Synonymity: n/a, Age × Synonymity: n.s.	n/a
Wiese et al., 2017 [14]	Exp 3: 20; 20	68; 23	50; 60	Semantic judgment	Name followed by a target face	32	300–600 & 400–500, 500–600	- N400 amplitude: O = Y, related < unrelated, Age × Relatedness: n.s., Age × Relatedness × Site (in the 400–500 ms time-window, relatedness effect at central and parietal sites in Y. but no relatedness effect in O)	- Accuracy: O = Y, related > unrelated, Age × Relatedness: n.s. - RTs: O > Y, related < unrelated, Age × Relatedness: n.s. (when considering the overall longer RTs in O)
Xu et al., 2017 [117]	24; 24	68; 22	50; 58	Semantic judgment	Sentences	64	50 ms time-windows from 250 to 550 ms after target onset	- N400 amplitude: O > Y between 250 and 300 ms, congruent < incongruent, Age × Congruence (smaller congruence effect in O vs. Y between 250 and 400 ms, driven by larger N400 for congruent stimuli in O), Age × Congruence × Region (450–500 ms: congruence effect in anterior and posterior regions for Y, and in posterior regions for O; 500–550 ms: no congruence effect in Y, congruence effect in posterior regions for O) - N400 effect peak latency: O > Y	- Accuracy: O = Y, congruent < incongruent, Age × Congruence (O < Y for incongruent stimuli only; congruent < incongruent in Y only) - RTs: n/a
Dave et al., 2018 [118]	36; 36	71; 21	58; 53	Sentence reading	Sentences	29	variable (100 ms time-window centered around N400 effect peak latency for each age group)	- N400 effect amplitude (context): O < Y	—
Dave et al., 2018 [44]	24; 24	72; 21	58; 54	Sentence reading	Sentences	29	Mean amplitude: 300–400, 350–450 and variable (100 ms time-window centered around N400 effect peak latency for each age group), peak latency: 150–650	- N400 amplitude: O = Y, low > moderate cloze, Age × Context (smaller context effect in O vs. Y between 350 and 450 ms), Age × Context × Anteriority × Hemisphere - N400 effect amplitude (context): O < Y - N400 effect peak latency (context): O > Y	—
Mah & Connolly, 2018 [119]	13; 26	70; 20	46; 73	Sentence and word listening	Sentences and word pairs	64	Mean amplitude: variable (50 ms time-window centered around N400 peak), peak latency: 300–700	- N400 amplitude: O < Y, congruent < low probability < incongruent sentences, Age × Condition: n.s. - N400 peak latency: O = Y, Condition: n.s. (difference for nonword condition only), Age × Sentence condition × Region	—
Zhu et al., 2018 [11]	20; 26	68; 22	45; 65	Semantic judgment	Sentences (targets in the middle)	62	Mean amplitude: O:350–550; Y: 300–500, peak latency: 250–600	- N400 amplitude: O = Y, congruent = incongruent, Age × Congruence (smaller congruence effect in O vs. Y), Age × Congruence × Region × Hemisphere (congruence effect in anterior and posterior regions over both hemispheres in Y, but over the right hemisphere only in O) - N400 effect peak latency: O > Y, Age × Region (O > Y in both anterior and posterior regions)	- Accuracy: O < Y, congruent = incongruent, Age × Congruence (semantic violation < semantic & syntactic violation in O only) - RTs: n/a
Cheimariou et al., 2019 [12]	21; 25	67; 24	48; 52	Semantic judgment	Picture-word pairs	32	250–500	- N400 amplitude: O < Y, congruent < incongruent, Age × Congruence: n.s., Age × Congruence × Predictiveness (congruence effect for strongly and weakly constraining conditions in O, congruence effect for the strongly constraining condition only in Y) - N400 fractional area latency: O > Y, Congruence: n.s., Age × Congruence (shorter for congruent vs. incongruent stimuli in O only)	- Accuracy: O = Y, congruent = incongruent, Age × Congruence: n/a - RTs: O > Y, congruent < incongruent, Age × Congruence: n/a
Federmeier & Kutas, 2019 [120]; Federmeier & Kutas, 1999 [121,122]	33; 36	67; 21	64; 47	Sentence reading	Sentences	26	350–550	- N400 effect amplitude: O < Y for prediction effect, and for congruence effect with right-visual field presentation	—
Kim & Jin, 2019 [123]	13; 13	69; 23	n/a; 0	Semantic judgment	Sentences	6 (P4, PZ, P3, C4, CZ, C3)	300–500	- N400 amplitude (implausible trials): O = Y - N400 peak latency (implausible trials): O = Y	- Accuracy: O < Y, Plausibility: n/a, Age × Plausibility: n/a - RTs: O > Y, Plausibility: n/a, Age × Plausibility: n/a
Lucas et al., 2019 [124]; Lucas et al., 2017 [125]	24; 24	69; 21	54; 75	Conceptual combination & frequency-comparison	Word pairs	29	300–500	- N400 effect amplitude (concreteness): O = Y	—
la Roi et al., 2020 [45]	25; 25	68; 22	40; 76	Sentence reading	Sentence pairs (context sentence, followed by the target-containing sentence; targets in the middle)	62	200–300, 300–400, 400–500	- N400 amplitude: Age: n/a, Correctness: n/a, predictive < neutral, Age × Correctness: n/a, Age × Context (predictive vs. neutral): n.s., Age × Correctness × Idiomaticity in the 3 time-windows (delayed correctness effect in O vs. Y)	—
Xu et al., 2020 [126]	43; 43	67; 22	56; 63	Semantic judgment	Sentences	64	O: 320–520; Y: 350–550	- N400 amplitude: O = Y, congruent < world knowledge violation < semantic violation, Age × Condition: n.s., Age × Condition × Region (congruent < world knowledge violation < semantic violation in posterior regions for O, congruent < both violation conditions in anterior and posterior regions for Y) - N400 effect peak latency: O > Y, semantic = world knowledge violation, Age × condition: n.s. - N400 effect onset latency: O > Y, semantic < world knowledge violation, Age × Condition (greater effect of condition in O vs. Y)	- Accuracy: O < Y, congruent & world knowledge violation < semantic violation, Age × Condition (congruent < violation conditions in Y only, semantic > world knowledge violation in O only) - RTs: n/a

^1^ We considered all tasks which required making a decision about the meaning of stimuli as semantic judgment tasks. ^2^ Unless specified otherwise, target stimuli were in final position (e.g., final words of a sentence). ^3^ We reported electrode sites for studies with ≤10 electrodes. ^4^ For the N400 amplitude and peak latency measures, we reported main effects of age/group and semantic variables and interaction effects involving both age/group and semantic variables in between-group analyses. For the N400 effect measures, we reported main effects of age/group and interactions involving the factor age/group. The interpretation of interactions is given in parenthesis when available. This table does not include results from N400 analyses performed on prime/context stimuli or investigating repetition effects nor results from additional analyses performed on individual electrode sites. For the sake of simplicity, when studies investigated the effects of lexical association with word pairs embedded in congruent and incongruent sentences, we only reported the effect of congruence at the sentence level. ^5^ Behavioral results are reported for studies that used semantic judgment tasks only (otherwise we indicated the symbol —). n/a: not tested or not reported, n.s.: not significant. O: Older; Y: Younger; AD: Alzheimer’s disease.

**Table A2:** Main characteristics of participants, materials and procedures and results of each study including AD participants with control group(s).

Reference	N of Subjects (AD; O; Y)	Mean Age (AD; O; Y)	Female % (AD; O; Y)	Task ^1^	Stimuli ^2^	N of Electrodes ^3^	N400 Time-Window (ms)	Main Results ^4^	
								N400	Behavior ^5^
Hamberger et al., 1995 [127]	(6; 10; 10)	(67; 67; 26)	(33; 30; 50)	Semantic judgment	Sentences	13	AD:425–525; O:400–500; Y:360–440	- N400 amplitude: AD = O = Y, best completion < unrelated/nonsense, Group × Word type (similar N400 gradient as a function of relatedness in AD and Y, and less graded pattern in O), Group × Word type × Hemisphere at T5/T6 electrode sites (Word type × Hemisphere in O only)	- Accuracy: AD < O & Y, best completion and unrelated/nonsense > related/sense and related/nonsense, Group × Relatedness: n.s. - RTs: AD > O > Y, Best completion < related/sense, Group × Relatedness (AD & Y: slowest RTs for related/nonsense, O: slowest RTs for related/sense)
Ford et al., 1996 [26]	(12; 12; 12)	(70; 70; 22)	(50; 50; 58)	Sentence listening	Sentences	13	Variable (300 ms time-window centered around peak latency)	- N400 amplitude: Group: yes (no post-hoc tests), congruent < incongruent, Group × Word type (larger N400 for incongruent trials in AD & Y vs. O; larger N400 for congruent trials in AD vs. Y & O) - N400 peak latency: AD = O, O = Y, congruent = incongruent, Group × Congruence: n.s.	—
Iragui et al., 1996 [17]	(12; 12; 12)	(70; 72; 24)	(33; 42; 33)	Semantic judgment	Phrases followed by a target word	13	Mean amplitude: 1rst analysis: 200–400, 400–600, 600–800, 2nd analysis: AD: 400–700; O: 300–600; Y: 200–500, 3rd analysis: 200–800, peak latency: 200–800	- N400 effect amplitude: 1rst analysis: Group: n/a, Group × Time-window; 2nd analysis: AD < O < Y, Group × Electrode (larger congruence effect over posterior vs. anterior sites in Y, less pronounced anterior-to-posterior gradient in O, no anterior-to-posterior gradient in AD); 3rd analysis: Group: n/a, Group × Stimulus type (greater congruence effect for opposite vs. categorical semantic relationships in Y only) - N400 effect peak and fractional area latencies: AD > O > Y	- Accuracy: AD < O & Y, Congruence: n/a, Group X Congruence (higher accuracy for congruent vs. incongruent stimuli in AD only) - RTs: n/a
Schwartz et al., 1996 [18]	(12; 12; 12)	(72; 73; 21)	(42; 75; 67)	Semantic judgment	Word pairs	13	Mean amplitude: 300–500, peak latency: Y:250–550; O:300–600; AD:400–700	- N400 effect amplitude: Group: yes (no post-hoc tests), Group × Hemisphere (Wernicke’s area: AD & O < Y, right-hemispheric homologue of Wernicke’s area: AD < O & Y, O = Y) - N400 effect peak latency: Group: n/a, Group × Hemisphere (AD > O > Y over the right-hemispheric homologue of Wernicke’s area only)	- Accuracy: AD < O & Y, Relatedness: n/a, subordinate and basic > superordinate level, Group × Relatedness: n/a - RTs: AD > O > Y, Relatedness: n/a, subordinate and basic < superordinate level, Group × Relatedness (relatedness effect in AD only)
Castañeda et al., 1997 [22]	(10; 10; -)	(75; 68; -)	(60; 50; -)	Semantic judgment	Picture pairs	32	300–580	- N400 amplitude: Group: n/a, congruent < incongruent, Group × Congruence (smaller congruence effect in AD vs. O, driven by smaller N400 for incongruent stimuli in AD vs. O) - N400 effect amplitude: AD < O - N400 effect peak latency: AD > O	- Accuracy: AD < Y (d’ & hits), Congruence: n/a, Group × Congruence: n/a - RTs: n/a
Ostrosky-Solís et al., 1998 [23]	(10; 10; 10)	(75; 68; 24)	(60; 50; 50)	Semantic judgment	Picture pairs	32	Mean amplitude: 325–550, N400 effect amplitude and peak latency: variable	- N400 amplitude: AD = O = Y, congruent < incongruent, Group × Congruence (smaller congruence effect in AD vs. O & Y, driven by smaller N400 for incongruent stimuli in AD vs. O & Y), Group × Congruence × Electrode (AD vs. Y: N400 differences at all sites, AD vs. O: N400 differences at Cz, Pz, Cp3, Cp4, T3, T4, Tp8, O vs. Y: N400 differences at Fz, Cz, Pz, Fc3, Cp3, T3, Tp7, Fc4, T4) - N400 effect amplitude: AD < O & Y, O = Y - N400 effect peak latency: AD = O, AD & O > Y	- Accuracy: AD < O & Y (d’ & hits), Congruence: n/a, Group × Congruence: n/a - RTs: n/a
Revonsuo et al., 1998 [70]	(9; 17; -)	(67; 67; -)	(67; 47; -)	Sentence listening and Semantic judgment after EEG recording	Sentences	20	300–800 & 100 ms time-windows from 200 to 1500 ms after target onset	- N400 amplitude: AD = O, congruent < incongruent, Group × Congruence: n.s. for the 300-800 ms time-window, but significant from 500 to 600 ms after target onset (smaller congruence effect in AD vs. O) - N400 amplitude (congruent trials only): AD = O	- Accuracy: AD < O, Congruence: n/a, Group × Congruence: n/a - RTs: n/a
Ford et al., 2001 [73]	(13; 13; 13)	(75; 74; 21)	(62; 62; 62)	Semantic judgment	Picture-word pairs	19	Variable (200 ms time-window centered around N400 effect peak latency)	- N400 amplitude: AD = O, O > Y, match < mismatch, Group/Age × Matching: n/a, Group (AD vs. O) × Matching × Laterality (matching × Laterality interaction in O only), Age × Matching × Anteriority (matching effect at central and parietal sites in O vs. at frontal, central and parietal sites in Y)	- Accuracy: Group (AD vs. O): n/a, O = Y, match = mismatch, Group/Age × Matching: n.s. - RTs: AD > O, O > Y, match < mismatch, Group/Age × Matching: n.s.
Auchterlonie et al., 2002 [128]	(8; 15; -)	(79; 71; -)	n/a	Semantic judgment	Word-picture pairs	17	n/a	- N400 amplitude: Group: n/a, Relatedness: n/a, Group × Relatedness: n/a, Group × Relatedness × Time (relatedness effect from 400 to 600 ms after target onset in O only)	- Accuracy: n/a - RTs: AD > O, related < unrelated, Group × Relatedness (greater relatedness effect in AD vs. O)
Schwartz et al., 2003 [129]	(12; 12; 12)	(77; 72; 24)	(58; 67; 50)	Semantic judgment	Sentences (targets in the middle for 1st and 2nd analyses, and in final position for the 3rd analysis)	15	1rst analysis: AD: 800–1000, O: 600–800; Y: 400–600, 2nd analysis: AD: 600–800; O: 400–600, 3rd analysis: 200–400, 400–600	- N400 amplitude: 1st analysis: Group: n.s., congruent < anomalous, Group × Congruence: n.s., 2nd analysis: AD < O, congruent-associated < anomalous-unassociated, Group × Congruence: n.s., 3rd analysis: Group: n/a, Congruence: n/a, Group × Congruence (smaller congruence effect in AD vs. Y from 200 to 400 ms after target onset, comparable congruence effect between groups from 400 to 600 ms)	- Accuracy: AD < O & Y, congruent < anomalous, Group × Congruence: n/a - RTs: AD > O & Y, Congruence: n/a, Group × Congruence: n/a
Wolk et al., 2005 [130]	(12; 12; -)	(72; 75; -)	(33; 50; -)	Recognition memory	Sentence stems followed by a target word	10 (FPz, Fz, Cz, Pz, Oz, F3, F4, P3, P4, Ml)	325–625	- N400 amplitude: AD > O, predictive = nonpredictive, Group × Context: n.s.	—
Olichney et al., 2006 [131]	(11; 11; -)	(79; 77; -)	(27; 36; -)	Semantic judgment	Phrases followed by a target word	≥15	300–550	- N400 amplitude: Group: n/a, congruent < incongruent, Group × Congruence: n.s. - N400 effect fractional area latency: AD = O	- Accuracy: AD < O, congruent = incongruent, Group × Congruence: n.s. - RTs: n/a
Taler et al., 2009 [132]	Exp 1: (10; 19; -)	(82; 75; -)	(70; 58; -)	Word reading	Word triplets	32	50 ms time-windows from 300 to 650 ms after target onset	- N400 amplitude: Group: n.s., related < unrelated, Group × Relatedness: n.s., Group × Relatedness × Time-windows at lateral electrode sites (relatedness effect from 500 to 650 ms after target onset in O; no relatedness effect in AD), Group × Ambiguity at lateral electrode sites (metonymic < metaphorical and homonymous stimuli in O only)	—
Bobes et al., 2010 [16]	(25; -; 27)	(48; -; 43)	n/a	Semantic judgment	Picture pairs	19	390–440	- N400 amplitude: Group: n.s., congruent < incongruent, Group × Congruence: n.s., Group × Congruence × Site - N400 effect amplitude: AD < Y at central, parietal temporal and occipital sites - N400 current source densities: Differences in AD vs. Y (e.g., decreased N400 sources in right temporal regions and increased N400 sources in the hippocampus and inferior temporal gyrus in the left hemisphere for AD vs. Y)	- Accuracy: AD < Y (d’) - RTs: n/a
Grieder et al., 2013 [72]	(19; 19; -)	(67; 70; -)	n/a	Lexical decision with semantic priming	Word pairs	128	Variable	- Topographic component recognition: N400 individual topographies of AD participants less spatially correlated with the group topography of the O vs. individual N400 topographies of the O	—

^1^ We considered all tasks which required making a decision about the meaning of stimuli as semantic judgment tasks. ^2^ Unless specified otherwise, target stimuli were in final position (e.g., final words of a sentence). ^3^ We reported electrode sites for studies with ≤10 electrodes. ^4^ For the N400 amplitude and peak latency measures, we reported main effects of age/group and semantic variables and interaction effects involving both age/group and semantic variables in between-group analyses. For the N400 effect measures, we reported main effects of age/group and interactions involving the factor age/group. The interpretation of interactions is given in parenthesis when available. This table does not include results from N400 analyses performed on prime/context stimuli or investigating repetition effects nor results from additional analyses performed on individual electrode sites. For the sake of simplicity, when studies investigated the effects of lexical association with word pairs embedded in congruent and incongruent sentences, we only reported the effect of congruence at the sentence level. ^5^ Behavioral results are reported for studies that used semantic judgment tasks only (otherwise we indicated the symbol —). n/a: not tested or not reported, n.s.: not significant. O: Older; Y: Younger; AD: Alzheimer’s disease.

## References

[1] Hoffman P. (2018). An individual differences approach to semantic cognition: Divergent effects of age on representation, retrieval and selection. Sci. Rep..

[2] Lambon Ralph M.A., Pobric G., Jefferies E. (2009). Conceptual knowledge is underpinned by the temporal pole bilaterally: Convergent evidence from rTMS. Cereb. Cortex.

[3] Ralph M.A., Jefferies E., Patterson K., Rogers T.T. (2017). The neural and computational bases of semantic cognition. Nat. Rev. Neurosci..

[4] Altmann L.J., McClung J.S. (2008). Effects of semantic impairment on language use in Alzheimer’s disease. Semin. Speech Lang..

[5] Verma M., Howard R.J. (2012). Semantic memory and language dysfunction in early Alzheimer’s disease: A review. Int. J. Geriatr. Psychiatry.

[6] Park D.C., Lautenschlager G., Hedden T., Davidson N.S., Smith A.D., Smith P.K. (2002). Models of visuospatial and verbal memory across the adult life span. Psychol. Aging.

[7] Toepper M. (2017). Dissociating Normal Aging from Alzheimer’s Disease: A View from Cognitive Neuroscience. J. Alzheimer’s Dis. JAD.

[8] Hoffman P., Morcom A.M. (2018). Age-related changes in the neural networks supporting semantic cognition: A meta-analysis of 47 functional neuroimaging studies. Neurosci. Biobehav. Rev..

[9] Chaby L., Jemel B., George N., Renault B., Fiori N. (2001). An ERP study of famous face incongruity detection in middle age. Brain Cogn..

[10] Miyamoto T., Katayama J., Koyama T. (1998). ERPs, semantic processing and age. Int. J. Psychophysiol..

[11] Zhu Z., Hou X., Yang Y. (2018). Reduced Syntactic Processing Efficiency in Older Adults During Sentence Comprehension. Front. Psychol..

[12] Cheimariou S., Farmer T.A., Gordon J.K. (2019). Lexical prediction in the aging brain: The effects of predictiveness and congruency on the N400 ERP component. Neuropsychol. Dev. Cogn. B Aging Neuropsychol. Cogn..

[13] Federmeier K.D., Van Petten C., Schwartz T.J., Kutas M. (2003). Sounds, words, sentences: Age-related changes across levels of language processing. Psychol. Aging.

[14] Wiese H., Komes J., Tuttenberg S., Leidinger J., Schweinberger S.R. (2017). Age-related differences in face recognition: Neural correlates of repetition and semantic priming in young and older adults. J. Exp. Psychol. Learn. Mem. Cogn..

[15] Kutas M., Federmeier K.D. (2011). Thirty years and counting: Finding meaning in the N400 component of the event-related brain potential (ERP). Annu. Rev. Psychol..

[16] Bobes M.A., Garcia Y.F., Lopera F., Quiroz Y.T., Galan L., Vega M., Trujillo N., Valdes-Sosa M., Valdes-Sosa P. (2010). ERP generator anomalies in presymptomatic carriers of the Alzheimer’s disease E280A PS-1 mutation. Hum. Brain Mapp..

[17] Iragui V., Kutas M., Salmon D.P. (1996). Event-related brain potentials during semantic categorization in normal aging and senile dementia of the Alzheimer’s type. Electroencephalogr. Clin. Neurophysiol..

[18] Schwartz T.J., Kutas M., Butters N., Paulsen J.S., Salmon D.P. (1996). Electrophysiological insights into the nature of the semantic deficit in Alzheimer’s disease. Neuropsychologia.

[19] Fabre L., Lemaire P. (2005). Age-related differences in automatic stimulus-response associations: Insights from young and older adults’ parity judgments. Psychon. Bull. Rev..

[20] Kutas M., Iragui V. (1998). The N400 in a semantic categorization task across 6 decades. Electroencephalogr. Clin. Neurophysiol..

[21] Wlotko E.W., Federmeier K.D., Kutas M. (2012). To predict or not to predict: Age-related differences in the use of sentential context. Psychol. Aging.

[22] Castaneda M., Ostrosky-Solis F., Perez M., Bobes M.A., Rangel L.E. (1997). ERP assessment of semantic memory in Alzheimer’s disease. Int. J. Psychophysiol..

[23] Ostrosky-Solis F., Castaneda M., Perez M., Castillo G., Bobes M.A. (1998). Cognitive brain activity in Alzheimer’s disease: Electrophysiological response during picture semantic categorization. J. Int. Neuropsychol. Soc..

[24] Gunter T.C., Jackson J.L., Mulder G. (1998). Priming and aging: An electrophysiological investigation of N400 and recall. Brain Lang..

[25] Federmeier K.D., Kutas M. (2005). Aging in context: Age-related changes in context use during language comprehension. Psychophysiology.

[26] Ford J.M., Woodward S.H., Sullivan E.V., Isaacks B.G., Tinklenberg J.R., Yesavage J.A., Roth W.T. (1996). N400 evidence of abnormal responses to speech in Alzheimer’s disease. Electroencephalogr. Clin. Neurophysiol..

[27] Ansado J., Marsolais Y., Methqal I., Alary F., Joanette Y. (2013). The adaptive aging brain: Evidence from the preservation of communication abilities with age. Eur. J. Neurosci..

[28] Moher D., Liberati A., Tetzlaff J., Altman D.G., Group P. (2009). Preferred reporting items for systematic reviews and meta-analyses: The PRISMA statement. PLoS Med..

[29] Egger M., Juni P., Bartlett C., Holenstein F., Sterne J. (2003). How important are comprehensive literature searches and the assessment of trial quality in systematic reviews? Empirical study. Health Technol. Assess..

[30] Mahood Q., Van Eerd D., Irvin E. (2014). Searching for grey literature for systematic reviews: Challenges and benefits. Res. Synth. Methods.

[31] Ali J.I., Smart C.M., Gawryluk J.R. (2018). Subjective Cognitive Decline and APOE varepsilon4: A Systematic Review. J. Alzheimer’s Dis. JAD.

[32] Keil A., Debener S., Gratton G., Junghofer M., Kappenman E.S., Luck S.J., Luu P., Miller G.A., Yee C.M. (2014). Committee report: Publication guidelines and recommendations for studies using electroencephalography and magnetoencephalography. Psychophysiology.

[33] Folstein M., Folstein S., McHugh P. (1975). Mini-mental state: A practical method of grading the cognitive state of patients for the clinician. J. Psychiatr. Res..

[34] Britt A.E., Ferrara C., Mirman D. (2016). Distinct Effects of Lexical and Semantic Competition during Picture Naming in Younger Adults, Older Adults, and People with Aphasia. Front. Psychol..

[35] Hertzog C., Raskind C.L., Cannon C.J. (1986). Age-related slowing in semantic information processing speed: An individual differences analysis. J. Gerontol..

[36] Pistono A., Busigny T., Jucla M., Cabirol A., Dinnat A.L., Pariente J., Barbeau E.J. (2019). An Analysis of Famous Person Semantic Memory in Aging. Exp. Aging Res..

[37] Mueller S.T., Piper B.J. (2014). The Psychology Experiment Building Language (PEBL) and PEBL Test Battery. J. Neurosci. Methods.

[38] Sleimen-Malkoun R., Temprado J.J., Berton E. (2013). Age-related dedifferentiation of cognitive and motor slowing: Insight from the comparison of Hick-Hyman and Fitts’ laws. Front. Aging Neurosci..

[39] Bilodeau-Mercure M., Kirouac V., Langlois N., Ouellet C., Gasse I., Tremblay P. (2015). Movement sequencing in normal aging: Speech, oro-facial, and finger movements. Age (Dordr).

[40] Hasher L., Zacks R.T. (1988). Working memory, comprehension and aging: A review and a new view. Psychol. Learn. Motiv..

[41] Humeau Y., Choquet D. (2019). The next generation of approaches to investigate the link between synaptic plasticity and learning. Nat. Neurosci..

[42] Schiera G., Di Liegro C.M., Di Liegro I. (2019). Cell-to-Cell Communication in Learning and Memory: From Neuro- and Glio-Transmission to Information Exchange Mediated by Extracellular Vesicles. Int. J. Mol. Sci..

[43] Wlotko E.W., Federmeier K.D. (2012). Age-related changes in the impact of contextual strength on multiple aspects of sentence comprehension. Psychophysiology.

[44] Dave S., Brothers T.A., Traxler M.J., Ferreira F., Henderson J.M., Swaab T.Y. (2018). Electrophysiological evidence for preserved primacy of lexical prediction in aging. Neuropsychologia.

[45] la Roi A., Sprenger S.A., Hendriks P. (2020). Event-related potentials reveal increased dependency on linguistic context due to cognitive aging. J. Exp. Psychol. Learn. Mem. Cogn..

[46] Federmeier K.D., McLennan D.B., De Ochoa E., Kutas M. (2002). The impact of semantic memory organization and sentence context information on spoken language processing by younger and older adults: An ERP study. Psychophysiology.

[47] Lehrner J., Coutinho G., Mattos P., Moser D., Pfluger M., Gleiss A., Auff E., Dal-Bianco P., Pusswald G., Stogmann E. (2017). Semantic memory and depressive symptoms in patients with subjective cognitive decline, mild cognitive impairment, and Alzheimer’s disease. Int. Psychogeriatr..

[48] Nebes R.D. (1989). Semantic memory in Alzheimer’s disease. Psychol. Bull..

[49] Cervera-Crespo T., Gonzalez-Alvarez J. (2017). Age and Semantic Inhibition Measured by the Hayling Task: A Meta-Analysis. Arch. Clin. Neuropsychol..

[50] Salehi M., Reisi M., Ghasisin L. (2017). Lexical Retrieval or Semantic Knowledge? Which One Causes Naming Errors in Patients with Mild and Moderate Alzheimer’s Disease?. Dement. Geriatr. Cogn. Dis. Extra.

[51] Myerson J., Lawrence B., Hale S., Jenkins L., Chen J. (1998). General slowing of lexical and nonlexical information processing in dementia of the Alzheimer type. Neuropsychol. Dev. Cogn. B Aging Neuropsychol. Cogn..

[52] Laisney M., Giffard B., Belliard S., de la Sayette V., Desgranges B., Eustache F. (2011). When the zebra loses its stripes: Semantic priming in early Alzheimer’s disease and semantic dementia. Cortex J. Devoted Study Nerv. Syst. Behav..

[53] Simoes Loureiro I., Lefebvre L. (2016). Distinct progression of the deterioration of thematic and taxonomic links in natural and manufactured objects in Alzheimer’s disease. Neuropsychologia.

[54] Lau E.F., Weber K., Gramfort A., Hamalainen M.S., Kuperberg G.R. (2016). Spatiotemporal Signatures of Lexical-Semantic Prediction. Cereb. Cortex.

[55] McCarthy G., Nobre A.C., Bentin S., Spencer D.D. (1995). Language-related field potentials in the anterior-medial temporal lobe: I. Intracranial distribution and neural generators. J. Neurosci..

[56] Nobre A.C., McCarthy G. (1995). Language-related field potentials in the anterior-medial temporal lobe: II. Effects of word type and semantic priming. J. Neurosci..

[57] Van Petten C., Luka B.J. (2006). Neural localization of semantic context effects in electromagnetic and hemodynamic studies. Brain Lang..

[58] Verclytte S., Lopes R., Lenfant P., Rollin A., Semah F., Leclerc X., Pasquier F., Delmaire C. (2016). Cerebral Hypoperfusion and Hypometabolism Detected by Arterial Spin Labeling MRI and FDG-PET in Early-Onset Alzheimer’s Disease. J. Neuroimaging.

[59] Huang J., Friedland R.P., Auchus A.P. (2007). Diffusion tensor imaging of normal-appearing white matter in mild cognitive impairment and early Alzheimer disease: Preliminary evidence of axonal degeneration in the temporal lobe. AJNR Am. J. Neuroradiol..

[60] Migliaccio R., Agosta F., Possin K.L., Canu E., Filippi M., Rabinovici G.D., Rosen H.J., Miller B.L., Gorno-Tempini M.L. (2015). Mapping the Progression of Atrophy in Early- and Late-Onset Alzheimer’s Disease. J. Alzheimer’s Dis. JAD.

[61] Bejanin A., Schonhaut D.R., La Joie R., Kramer J.H., Baker S.L., Sosa N., Ayakta N., Cantwell A., Janabi M., Lauriola M. (2017). Tau pathology and neurodegeneration contribute to cognitive impairment in Alzheimer’s disease. Brain.

[62] Kang S.H., Park Y.H., Lee D., Kim J.P., Chin J., Ahn Y., Park S.B., Kim H.J., Jang H., Jung Y.H. (2019). The Cortical Neuroanatomy Related to Specific Neuropsychological Deficits in Alzheimer’s Continuum. Dement. Neurocogn. Disord..

[63] Melrose R.J., Campa O.M., Harwood D.G., Osato S., Mandelkern M.A., Sultzer D.L. (2009). The neural correlates of naming and fluency deficits in Alzheimer’s disease: An FDG-PET study. Int. J. Geriatr. Psychiatry.

[64] Zahn R., Juengling F., Bubrowski P., Jost E., Dykierek P., Talazko J., Huell M. (2004). Hemispheric asymmetries of hypometabolism associated with semantic memory impairment in Alzheimer’s disease: A study using positron emission tomography with fluorodeoxyglucose-F18. Psychiatry Res..

[65] Kirova A.M., Bays R.B., Lagalwar S. (2015). Working memory and executive function decline across normal aging, mild cognitive impairment, and Alzheimer’s disease. Biomed. Res. Int..

[66] Holcomb P.J. (1988). Automatic and attentional processing: An event-related brain potential analysis of semantic priming. Brain Lang..

[67] Kiefer M., Brendel D. (2006). Attentional modulation of unconscious “automatic” processes: Evidence from event-related potentials in a masked priming paradigm. J. Cogn. Neurosci..

[68] Kim A.E., Oines L., Miyake A. (2018). Individual differences in verbal working memory underlie a tradeoff between semantic and structural processing difficulty during language comprehension: An ERP investigation. J. Exp. Psychol. Learn. Mem. Cogn..

[69] Wechsler D. (1955). Manual for the Wechsler Adult Intelligence Scale.

[70] Revonsuo A., Portin R., Juottonen K., Rinne J.O. (1998). Semantic processing of spoken words in Alzheimer’s disease: An electrophysiological study. J. Cogn. Neurosci..

[71] Kaplan E., Goodglass H., Weintraub S. (1983). Boston Naming Test.

[72] Grieder M., Crinelli R.M., Jann K., Federspiel A., Wirth M., Koenig T., Stein M., Wahlund L.O., Dierks T. (2013). Correlation between topographic N400 anomalies and reduced cerebral blood flow in the anterior temporal lobes of patients with dementia. J. Alzheimer’s Dis. JAD.

[73] Ford J.M., Askari N., Mathalon D.H., Menon V., Gabrieli J.D., Tinklenberg J.R., Yesavage J. (2001). Event-related brain potential evidence of spared knowledge in Alzheimer’s disease. Psychol. Aging.

[74] Spreng R.N., Turner G.R. (2019). The Shifting Architecture of Cognition and Brain Function in Older Adulthood. Perspect. Psychol. Sci..

[75] Schaie K.W. (1994). The course of adult intellectual development. Am. Psychol..

[76] Kaufman A.S. (2001). WAIS-III IQs, Horn’s theory, and generational changes from young adulthood to old age. Intelligence.

[77] Clayson P.E., Carbine K.A., Baldwin S.A., Larson M.J. (2019). Methodological reporting behavior, sample sizes, and statistical power in studies of event-related potentials: Barriers to reproducibility and replicability. Psychophysiology.

[78] Larson M.J., Carbine K.A. (2017). Sample size calculations in human electrophysiology (EEG and ERP) studies: A systematic review and recommendations for increased rigor. Int. J. Psychophysiol..

[79] Duncan C.C., Barry R.J., Connolly J.F., Fischer C., Michie P.T., Naatanen R., Polich J., Reinvang I., Van Petten C. (2009). Event-related potentials in clinical research: Guidelines for eliciting, recording, and quantifying mismatch negativity, P300, and N400. Clin. Neurophysiol. Off. J. Int. Fed. Clin. Neurophysiol..

[80] Tanner D., Morgan-Short K., Luck S.J. (2015). How inappropriate high-pass filters can produce artifactual effects and incorrect conclusions in ERP studies of language and cognition. Psychophysiology.

[81] Holcomb P.J., Grainger J., O’Rourke T. (2002). An electrophysiological study of the effects of orthographic neighborhood size on printed word perception. J. Cogn. Neurosci..

[82] Swaab T.Y., Baynes K., Knight R.T. (2002). Separable effects of priming and imageability on word processing: An ERP study. Brain Res. Cogn. Brain Res..

[83] West W.C., Holcomb P.J. (2000). Imaginal, semantic, and surface-level processing of concrete and abstract words: An electrophysiological investigation. J. Cogn. Neurosci..

[84] Barton J.J., Hanif H.M., Eklinder Bjornstrom L., Hills C. (2014). The word-length effect in reading: A review. Cogn. Neuropsychol..

[85] Huang H.W., Meyer A.M., Federmeier K.D. (2012). A “concrete view” of aging: Event related potentials reveal age-related changes in basic integrative processes in language. Neuropsychologia.

[86] Wilkinson A.J., Yang L., Dyson B.J. (2013). Modulating younger and older adults’ performance in ignoring pictorial information during a word matching task. Brain Cogn..

[87] DeLong K.A., Quante L., Kutas M. (2014). Predictability, plausibility, and two late ERP positivities during written sentence comprehension. Neuropsychologia.

[88] Lau E.F., Namyst A. (2019). fMRI evidence that left posterior temporal cortex contributes to N400 effects of predictability independent of congruity. Brain Lang..

[89] Harbin T.J., Marsh G.R., Harvey M.T. (1984). Differences in the late components of the event-related potential due to age and to semantic and non-semantic tasks. Electroencephalogr. Clin. Neurophysiol..

[90] Gunter T.C., Jackson J.L., Mulder G. (1992). An electrophysiological study of semantic processing in young and middle-aged academics. Psychophysiology.

[91] Hamberger M., Friedman D. (1992). Event-related potential correlates of repetition priming and stimulus classification in young, middle-aged, and older adults. J. Gerontol..

[92] Gunter T.C., Jackson J.L., Mulder G. (1995). Language, memory, and aging: An electrophysiological exploration of the N400 during reading of memory-demanding sentences. Psychophysiology.

[93] Gunter T.C., Jackson J.L., Mulder G. (1996). Focussing on aging: An electrophysiological exploration of spatial and attentional processing during reading. Biol Psychol..

[94] Cameli L., Phillips N.A. (2000). Age-related differences in semantic priming: Evidence from event-related brain potentials. Brain Cogn..

[95] Bonnaud V., Gil R., Ingrand P. (2002). Metaphorical and non-metaphorical links: A behavioral and ERP study in young and elderly adults. Neurophysiol. Clin..

[96] Phillips N.A., Lesperance D. (2003). Breaking the waves: Age differences in electrical brain activity when reading text with distractors. Psychol. Aging.

[97] Nessler D., Johnson R., Bersick M., Friedman D. (2006). On why the elderly have normal semantic retrieval but deficient episodic encoding: A study of left inferior frontal ERP activity. Neuroimage.

[98] Faustmann A., Murdoch B.E., Finnigan S.P., Copland D.A. (2007). Effects of advancing age on the processing of semantic anomalies in adults: Evidence from event-related brain potentials. Exp. Aging Res..

[99] Federmeier K.D., Kutas M., Schul R. (2010). Age-related and individual differences in the use of prediction during language comprehension. Brain Lang..

[100] Kawohl W., Bunse S., Willmes K., Hoffrogge A., Buchner H., Huber W. (2010). Semantic event-related potential components reflect severity of comprehension deficits in aphasia. Neurorehabil. Neural Repair.

[101] Kousaie S., Phillips N.A. (2011). Age-related differences in interlingual priming: A behavioural and electrophysiological investigation. Neuropsychol. Dev. Cogn. B Aging Neuropsychol. Cogn..

[102] Lee C.L., Federmeier K.D. (2009). Wave-ering: An ERP study of syntactic and semantic context effects on ambiguity resolution for noun/verb homographs. J. Mem. Lang..

[103] Lee C.L., Federmeier K.D. (2011). Differential age effects on lexical ambiguity resolution mechanisms. Psychophysiology.

[104] Grieder M., Crinelli R.M., Koenig T., Wahlund L.O., Dierks T., Wirth M. (2012). Electrophysiological and behavioral correlates of stable automatic semantic retrieval in aging. Neuropsychologia.

[105] Huang H.W., Lee C.L., Federmeier K.D. (2010). Imagine that! ERPs provide evidence for distinct hemispheric contributions to the processing of concrete and abstract concepts. Neuroimage.

[106] Lee C.L., Federmeier K.D. (2012). Ambiguity’s aftermath: How age differences in resolving lexical ambiguity affect subsequent comprehension. Neuropsychologia.

[107] Davis T.M., Jerger J., Martin J. (2013). Electrophysiological evidence of augmented interaural asymmetry in middle-aged listeners. J. Am. Acad. Audiol..

[108] Molnar M., Toth B., Boha R., Gaal Z.A., Kardos Z., File B., Stam C.J. (2013). Aging effects on ERP correlates of emotional word discrimination. Clin. Neurophysiol. Off. J. Int. Fed. Clin. Neurophysiol..

[109] Mehta J., Jerger J. (2014). Variation in semantic priming across age groups: An AERP study. Int. J. Audiol..

[110] Zhou T., Li J., Broster L.S., Niu Y., Wang P. (2015). Reduced late positivity in younger adults, but not older adults, during short-term repetition. Brain Res..

[111] Chang C.T., Lee C.Y., Chou C.J., Fuh J.L., Wu H.C. (2016). Predictability effect on N400 reflects the severity of reading comprehension deficits in aphasia. Neuropsychologia.

[112] Ghosh Hajra S., Liu C.C., Song X., Fickling S., Liu L.E., Pawlowski G., Jorgensen J.K., Smith A.M., Schnaider-Beeri M., Van Den Broek R. (2016). Developing Brain Vital Signs: Initial Framework for Monitoring Brain Function Changes Over Time. Front. Neurosci..

[113] Khachatryan E., De Letter M., Vanhoof G., Goeleven A., Van Hulle M.M. (2016). Sentence Context Prevails Over Word Association in Aphasia Patients with Spared Comprehension: Evidence from N400 Event-Related Potential. Front. Hum. Neurosci..

[114] Payne B.R., Federmeier K.D. (2017). Event-related brain potentials reveal age-related changes in parafoveal-foveal integration during sentence processing. Neuropsychologia.

[115] Stites M.C., Payne B.R., Federmeier K.D. (2017). Getting ahead of yourself: Parafoveal word expectancy modulates the N400 during sentence reading. Cogn. Affect. Behav. Neurosci..

[116] Weißbecker-Klaus X., Ullsperger P., Freude G., Schapkin S.A. (2017). Impaired error processing and semantic processing during multitasking. J. Psychophysiol..

[117] Xu N., Hou X., Zhao B., Zhu Z., Yang Y. (2017). Age-related temporal-spatial dynamic ERP changes during sentence comprehension. Neurosci. Lett..

[118] Dave S., Brothers T.A., Swaab T.Y. (2018). 1/f neural noise and electrophysiological indices of contextual prediction in aging. Brain Res..

[119] Mah R.L., Connolly J.F. (2018). A framework for the extended monitoring of levels of cognitive function in unresponsive patients. PLoS ONE.

[120] Federmeier K.D., Kutas M. (2019). What’s “left”? Hemispheric sensitivity to predictability and congruity during sentence reading by older adults. Neuropsychologia.

[121] Federmeier K.D., Kutas M. (1999). Right words and left words: Electrophysiological evidence for hemispheric differences in meaning processing. Brain Res. Cogn. Brain Res..

[122] Federmeier K.D., Kutas M. (1999). A Rose by Any Other Name: Long-Term Memory Structure and Sentence Processing. J. Mem. Lang..

[123] Kim H., Jin Y. (2019). Emotion and reading comprehension in elderly and young adults: An ERP study. NeuroQuantology.

[124] Lucas H.D., Gupta R.S., Hubbard R.J., Federmeier K.D. (2019). Adult Age Differences in the Use of Conceptual Combination as an Associative Encoding Strategy. Front. Hum. Neurosci..

[125] Lucas H.D., Hubbard R.J., Federmeier K.D. (2017). Flexible conceptual combination: Electrophysiological correlates and consequences for associative memory. Psychophysiology.

[126] Xu N., Chen S., Yang Y., Zhu Z. (2020). Increased world knowledge in older adults does not prevent decline in world knowledge comprehension: An ERP study. Brain Cogn..

[127] Hamberger M.J., Friedman D., Ritter W., Rosen J. (1995). Event-related potential and behavioral correlates of semantic processing in Alzheimer’s patients and normal controls. Brain Lang..

[128] Auchterlonie S., Phillips N.A., Chertkow H. (2002). Behavioral and electrical brain measures of semantic priming in patients with Alzheimer’s disease: Implications for access failure versus deterioration hypotheses. Brain Cogn..

[129] Schwartz T.J., Federmeier K.D., Van Petten C., Salmon D.P., Kutas M. (2003). Electrophysiological analysis of context effects in Alzheimer’s disease. Neuropsychology.

[130] Wolk D.A., Schacter D.L., Berman A.R., Holcomb P.J., Daffner K.R., Budson A.E. (2005). Patients with mild Alzheimer’s disease attribute conceptual fluency to prior experience. Neuropsychologia.

[131] Olichney J.M., Iragui V.J., Salmon D.P., Riggins B.R., Morris S.K., Kutas M. (2006). Absent event-related potential (ERP) word repetition effects in mild Alzheimer’s disease. Clin. Neurophysiol. Off. J. Int. Fed. Clin. Neurophysiol..

[132] Taler V., Klepousniotou E., Phillips N.A. (2009). Comprehension of lexical ambiguity in healthy aging, mild cognitive impairment, and mild Alzheimer’s disease. Neuropsychologia.

